# Synovial macrophage rhoa protects against osteoarthritis by suppressing YAP/IL-17C mediated chondrocyte senescence

**DOI:** 10.1007/s10565-026-10151-w

**Published:** 2026-01-31

**Authors:** Yizhou Xu, Shuyi Xu, Jiayi Li, Jiaqi Wang, Jie Liang, Jiale Cai, Xianghai Wang, Ying Zou, Gang Deng, Jiasong Guo, Lixin Zhu

**Affiliations:** 1 Department of Spinal Surgery, Orthopedic Medical Center, Zhujiang Hospital, Southern Medical University, Guangzhou, 510280 China; 2 Department of Histology and Embryology, School of Basic Medical Sciences, Southern Medical University, Guangzhou, 510515 China; 3 Department of Sport Medicine, Ganzhou Hospital-Nanfang Hospital, Southern Medical University, Ganzhou, 341000 China

**Keywords:** Osteoarthritis, RhoA, Synovial Macrophage, Chondrocyte Senescence, YAP/IL-17C

## Abstract

**Objective:**

The GTPase RhoA is known as a regulator involved in cartilage degeneration and subchondral bone remodeling related to osteoarthritis (OA). However, its specific role in synovial macrophages, the key immune cells of OA related tissues, remains entirely unexplored.

**Methods:**

Herein, the RhoA expression in human and mouse OA synovium was analyzed. A macrophage-specific RhoA conditional knockout (cKO) mouse model was generated. Histological staining, OARSI scoring, and micro-CT were used to assess cartilage damage, while Western blot, immunofluorescence staining, and ELISA assessed changes in cellular function. Transcriptome sequencing and validation of signaling pathways were conducted using tissues and cells from patients with OA and OA mice.

**Results:**

The collected results indicate that RhoA expression was significantly upregulated in synovial macrophages from OA patients and mice, correlating with disease severity. Contrary to its reported role in chondrocytes or endothelial cells, macrophage-specific RhoA deletion exacerbated OA, demonstrating enhanced cartilage destruction, subchondral bone loss, and synovitis. RhoA-deficient macrophages exhibited a pro-inflammatory M1 polarization and secreted high levels of IL-17C. This cytokine was necessary and sufficient to induce chondrocyte senescence, as evidenced by increased p53/p21, ROS, mitochondrial dysfunction, and suppressed autophagy, via activation of the PI3K/AKT/mTOR pathway. Mechanistically, RhoA ablation in macrophages activated the Hippo pathway effectors YAP/CCN2, leading to IL-17C transcription, independently of the canonical ROCK pathway.

**Conclusion:**

In conclusion, present study reveals a previously unrecognized, protective role for macrophage RhoA in OA. It functions as a critical brake on a novel YAP-IL-17C axis, thereby preserving chondrocyte. This study redefines RhoA's role in joint homeostasis and nominates IL-17C as a potential therapeutic target for OA.

**Graphical Abstract:**

**Figure:**
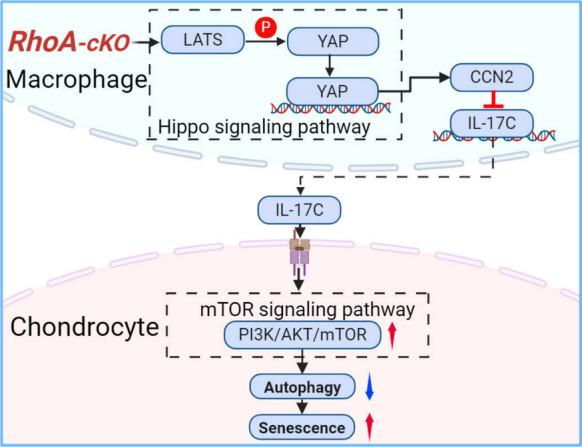
1. Macrophage RhoA deficiency accelerates OA progression 2. RhoA knockout in macrophages enhances IL-17C secretion through YAP/CCN2 pathway 3. IL-17C triggers chondrocyte senescence via PI3K/AKT/mTOR signaling

**Supplementary Information:**

The online version contains supplementary material available at 10.1007/s10565-026-10151-w.

## Introduction

Osteoarthritis (OA) is a highly prevalent and debilitating degenerative joint disease, affecting 600 million people worldwide (Duong et al. 2023; Hunter and Bierma-Zeinstra 2019; Roelofs and De Bari 2024). OA is well known as its progressive synovial inflammation, cartilage deterioration, and subchondral bone remodeling, however, the molecular drivers of OA pathogenesis remain incompletely elucidated, hindering the development of efficient therapeutic strategies (Jia et al. 2024; Kloppenburg 2023; Xu et al. 2023). Among the various signaling pathways implicated in OA, the Rho GTPase RhoA has garnered increasing attention (Deng et al. 2019; Novakofski et al. 2009). Accumulating evidence positions RhoA as a central regulator of cytoskeletal dynamics, cell differentiation, and inflammatory responses within the joint. Studies have documented its pathogenic role in promoting chondrocyte hypertrophy, cartilage matrix degradation, and vascular dysfunction in subchondral bone, highlighting RhoA as a potential therapeutic target (Sui et al. 2022; Wang and Beier 2005; Wang et al. 2004; Zhang et al. 2025).

However, the existing researches primarily focused on RhoA functions in chondrocytes and endothelial cells (He et al. 2024; Jiang et al. 2023). The role of RhoA in synovial macrophages, key sentinels and amplifiers of joint inflammation, has never been reported. Given that macrophage polarization critically influences OA progression (Cai et al. 2025; Jiang et al. 2025; Wang et al. 2025), we hypothesized that RhoA signaling within these immune cells could be a pivotal regulator of the synovial environment and cartilage homeostasis.

In this study, we systematically investigated the expression and functional significance of RhoA in synovial macrophages during OA. Combined with clinical OA samples, experimental OA model in wild type mice and macrophage-specific conditional knockout (cKO) mice, and in vitro culture systems, the present findings uncovered an unexpected role of that RhoA upregulation in OA synovial macrophages exerts a protective, cell-type-specific function in OA. Mechanically, macrophages RhoA modulates IL-17C secretion through the non-canonical Hippo/YAP signaling pathway, and the IL-17C paracrine drives chondrocyte senescence via the PI3K/AKT/mTOR pathway. Present study not only broaden the understanding of RhoA in OA but also reveal a previously unrecognized crosstalk mechanism between synovial macrophages and articular chondrocytes.

## Materials and methods

### Experimental animals and human OA samples

#### Animals

Male C57BL/6 mice (8 weeks, 20–22 g, SPF) were purchased from the Southern Medical University Animal Center (Guangzhou, China) and maintained at 22 ± 2 °C, 55 ± 10% humidity and a 12-h light/dark cycle. OA was induced by destabilization of the medial meniscus (DMM) at 8 weeks of age. For primary chondrocyte isolation, 3-day-old neonatal pups were euthanized by decapitation. All procedures were approved by the Institutional Animal Care and Use Committee of Southern Medical University (approval no. SMU-L2018158).

#### Transgenic mice

Lyz2-Cre mice (Sangon Biotech, Suzhou, China) were backcrossed to C57BL/6 for ≥ 6 generations and then crossed with RhoA^flox/flox^ mice to produce macrophage-specific (Lyz2-Cre; RhoA^flox/flox^) RhoA knockouts. Cre-negative RhoA^flox/flox^ littermates served as controls. Genotyping was performed following the protocols described in our previous publication (Xu et al. 2021).

#### Human tissues

Synovial samples were obtained from patients with end-stage OA (KL grade 1–4; mean age 67 ± 5) undergoing total knee replacement and from trauma-amputation controls (mean age 35 ± 10) at the Ganzhou Hospital–Nanfang Hospital. The study protocol was approved by the Institutional Review Board (approval no. TY-ZKY2024-141–01), and written informed consent was obtained from all participants.

### Primary cell and OA cartilage culture

Primary chondrocytes were isolated from 3-day-old C57BL/6 pups by enzymatic digestion (0.2% type II Collagenase, 2 h, 37 °C) and cultured in DMEM/F-12 with 10% FBS and 1% penicillin–streptomycin; cells were used at passage ≤ 2. Bone marrow–derived macrophages (BMDMs) were generated by flushing femora and tibiae from 8-week-old mice and culturing in DMEM containing 10% FBS and 20 ng/mL recombinant murine M-CSF (PeproTech, 315–02) for 7 days; purity was > 95% (Zou et al. 2021).

Human OA cartilage was obtained from total-knee-replacement patients within 2 h of surgery, minced into 1 cm^2^ explants and cultured in DMEM/F-12 plus 10% FBS at 37 °C, 5% CO₂ (Xu et al. 2023). All human tissues were collected under approval TY-ZKY2024-141–01 with written informed consent.

### Animal surgery and drug administration

OA was induced by destabilisation of the medial meniscus (DMM) in the right knee of 8-week-old male C57BL/6 mice. Under sterile conditions and continuous isoflurane anaesthesia (2% in O₂, 1 L/min), the medial meniscotibial ligament was transected under a dissecting microscope (× 10). Sham mice underwent identical arthrotomy without ligament transection (Xu et al. 2023). Post-operative analgesia (Carprofen 5 mg/kg s.c. every 12 h for 48 h) and prophylactic antibiotics (enrofloxacin 5 mg/kg s.c. once daily for 3 days) were administered.

Beginning 7 days after surgery, mice received weekly intra-articular injections (30-gauge needle via the patellar tendon, 9–11 a.m.) for 7 consecutive weeks: (1) DMM + CT04: CT04 (1 μg/μL, 10 μL (Zhang et al. 2012)) in 0.9% NaCl, pH 7.4. (2) DMM + CT04 + Anti-IL-17C: CT04 (2 μg/μL, 5 μL) plus Anti-IL-17C (20 mg/kg, 5 μL in 0.9% NaCl (Zhou et al. 2020b)). (3) DMM + vehicle (Control group): equal volume of sterile 0.9% NaCl. Stock solutions were sterile-filtered (0.22 μm), aliquoted, and stored at − 80 °C. Aliquots were thawed at 4 °C immediately before use, vortexed for 5 s, centrifuged 1 min at 4 °C, and kept on ice ≤ 2 h prior to injection.

### Micro-computed tomography

The mouse knee tissues were preserved in a 4% paraformaldehyde solution for 72 h before undergoing micro-computed tomography (micro-CT) analysis, following established methodologies. The analysis assessed several parameters, including the osteophyte count, trabecular separation (Tb. Sp, mm), trabecular thickness (Tb.Th, mm), and trabecular number (Tb. N, 1/mm). These parameters were quantified using the accompanying software for the micro-CT system (ZKKS-MicroCT4.1, China).

### Histological staining

Knee joints were fixed in 4% paraformaldehyde for 24 h at 4 °C, decalcified in 10% (w/v) EDTA (pH 7.4, 4 °C, 50 days), dehydrated through ascending ethanol (70%, 80%, 90%, 100%, each 30 min), embedded in paraffin and sectioned into 5 μm.

Safranin O-fast green (0.1% Safranin O, 5 min; 0.2% Fast Green, 5 min) and Toluidine blue (0.04%, 2 min) staining were performed. Cartilage degeneration was scored according to OARSI 2019 guidelines (0–6 scale) by two blinded observers. Synovitis was graded on H&E-stained Sects. (0–9 scale).

For immunohistochemistry, sections were deparaffinized, rehydrated, subjected to heat-mediated antigen retrieval (citrate buffer, pH 6.0, 95 °C, 20 min), incubated with 3% H₂O₂ (15 min) and blocked with goat serum (30 min). Primary antibodies (dilutions in Table S1) were incubated overnight at 4 °C, followed by HRP-conjugated secondary antibody at room temperature (RT, 1 h) and DAB (≤ 3 min). Sections were counterstained with haematoxylin, dehydrated and mounted.

For immunofluorescence, antigen retrieval and blocking (5% normal goat serum, 1 h) were performed as above. Primary antibodies were incubated overnight at 4 °C, followed by Alexa Fluor-conjugated secondary antibodies (488 or 568, 2 h, RT). Nuclei were stained with DAPI (1 μg/mL, 5 min). Images were captured using a Nikon A1 confocal microscope at identical settings. The list of antibodies used is provided in the Supplementary Materials Table 1.

### RNA sequencing

Total RNA was extracted using Trizol Reagent (Invitrogen Life Technologies), with concentration, quality, and integrity assessed via a NanoDrop spectrophotometer (Thermo Scientific). Three micrograms of RNA were used for library preparation. mRNA was isolated from total RNA using poly-T oligo-attached magnetic beads, followed by fragmentation using divalent cations in an Illumina proprietary buffer at elevated temperature. First-strand cDNA was synthesized with random primers and Super-Script II, and second-strand synthesis was performed using DNA Polymerase I and RNase H. Exonuclease/polymerase activities converted remaining overhangs into blunt ends, and the enzymes were removed. Following adenylation of the 3′ ends, Illumina PE adapter oligonucleotides were ligated for hybridization preparation. To select cDNA fragments of 400–500 bp, the library was purified using the AMPure XP system (Beckman Coulter, Beverly, CA, USA). Adapter-ligated DNA fragments were enriched through 15 cycles of PCR using Illumina PCR Primer Cocktail. The products were purified (AMPure XP system) and quantified using the Agilent high-sensitivity DNA assay on a Bioanalyzer 2100 system (Agilent). The sequencing library was processed on the NovaSeq 6000 platform (Illumina) at Shanghai Personal Biotechnology Cp. Ltd. Transcriptome analysis followed the manufacturer's protocol.

### Single-cell RNA sequencing data processing

The single-cell RNA sequencing dataset GSE133449 (Sun et al. 2020) (10.1136/annrheumdis-2019–215926) was processed using the Seurat R package. These data were originated from the patients with OA (*n* = 4). Initial quality control steps included filtering cells with high mitochondrial gene content (> 20%) and low gene count (< 200 genes per cell). Normalization and scaling of the data were performed, followed by the identification of highly variable genes across cells. A differential expression analysis was performed on macrophages cluster (MS4A7^+^) (Zhou et al. 2020a), and the result was visualized using Uniform Manifold Approximation and Projection (UMAP). MS4A7^+^ macrophages were colored according to its expression level of RhoA, allowing us to spatially observe the distribution of gene expression within the UMAP embedding.

### Western blotting

After washing with PBS, tissues and cells were collected in RIPA lysis buffer (Cat#FD009, Fdbio Science, China) containing phosphatase and protease inhibitors, followed by protein extraction through lysis at RT for 30 min. Proteins were separated by SDS-PAGE and transferred to polyvinylidene fluoride membranes (Cat#IPVH0010, Millipore, USA) according to standard protocols. Membranes were blocked with 5% skim milk for 1 h at RT, then cut horizontally based on molecular weight, as labelled by Precision Plus Protein (10—250 kD, Cat#1610374S, Bio-Rad, USA). Membranes were incubated with primary antibody at 4 °C overnight. To detect phosphorylated proteins at the same site, membranes were stripped using Stripping buffer (Cat#ab282569, Abcam, UK), re-blocked with 5% skim milk for 1 h at RT, and incubated with primary antibody overnight at 4 °C. On the following day, horseradish peroxidase (HRP)-conjugated secondary antibodies were applied, and enhanced chemiluminescence was used to detect target protein expression levels. Information on the antibodies utilized is provided in Supplementary materials Table 1.

### Statistical analyses

Statistical analysis was performed using SPSS 25.0 software. Comparisons were made using the t-test, one-way ANOVA, or two-way ANOVA, followed by Tukey's post hoc test. Data are presented as mean ± S.D. (n ≥ 3), with *P* < 0.05 considered statistically significant.

## Results

### RhoA is upregulated in synovial macrophages and correlates with OA severity

**Fig. 1:**
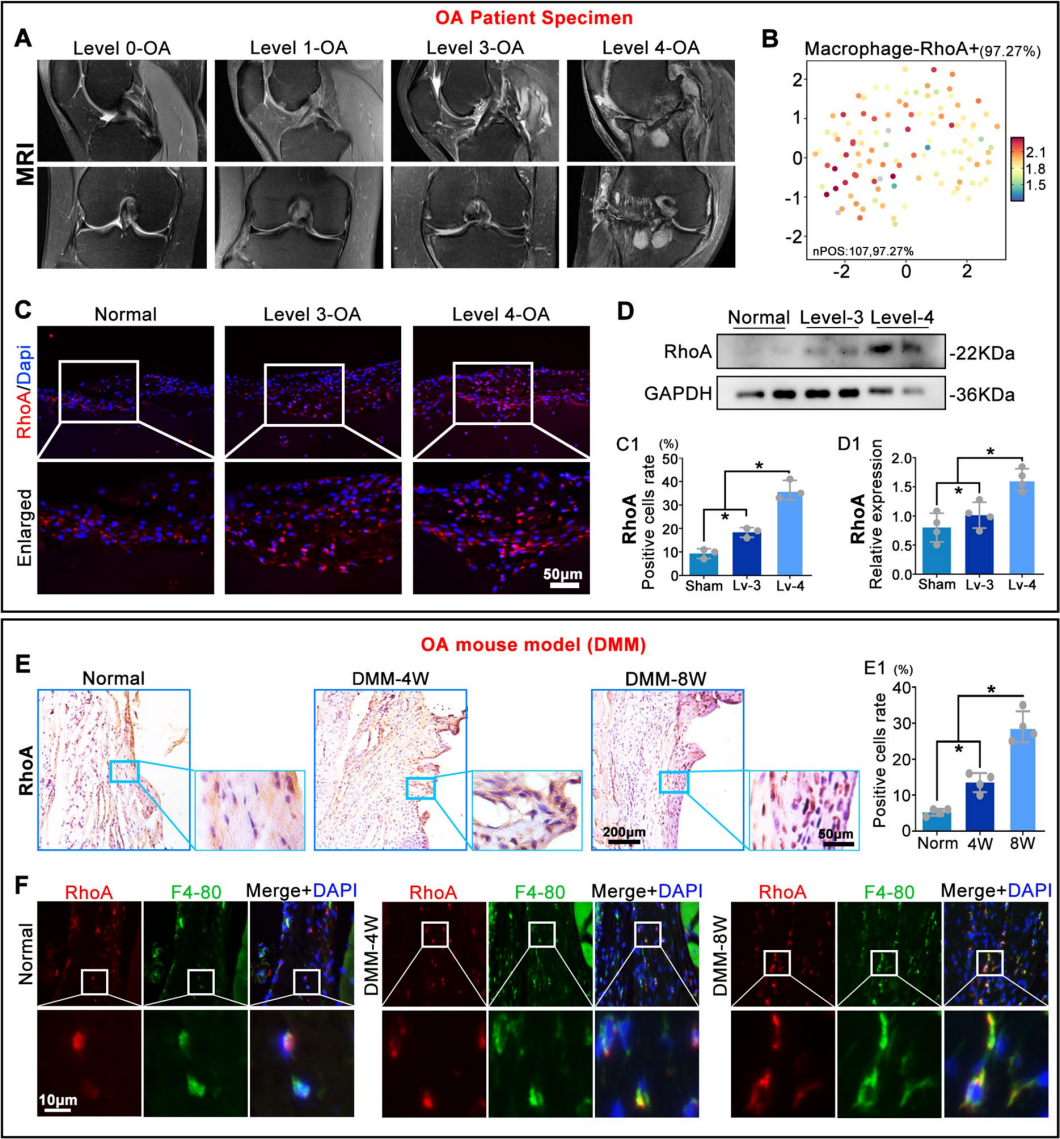
RhoA expression in OA patients and mouse models. **A**. Representative MRI images of OA patients. **B**. The expression level of RhoA in macrophages was visualized using UMAP. **C**,** D**. IF and WB analysis of RhoA of positive cells rate and protein expression in ex vivo human OA synovial tissues. **E**. IHC for RhoA in synovium: percentage of RhoA⁺ cells relative to total haematoxylin-stained nuclei. **F**. IF co-staining: percentage of RhoA⁺ F4/80⁺ double-positive cells relative to total DAPI⁺ nuclei. *P < 0.05

To investigate the role of macrophage RhoA in OA progression and its correlation with disease severity, synovial tissues were collected from OA patients during surgery. Samples were stratified based on OA severity determined by MRI grading (Fig. 1A). Bioinformatic analysis based on single-cell RNA sequencing data from the GEO database (GSE133449) indicated that RhoA was highly expressed in macrophages within OA tissues (Fig. 1B). Immunofluorescence (IF) and Western blot (WB) analyses in the human samples confirmed that RhoA positive cells rate and protein levels in synovial tissues increased with OA severity (Fig. 1C, D). In a mouse OA model, immunohistochemistry (IHC) revealed the RhoA positive intensity in the synovial is gradually increased at 4 and 8 weeks post-surgery (Fig. 1E). Furthermore, double immunofluorescent-staining identified that the RhoA expression in synovial is mainly localized in F4/80⁺ macrophages (Fig. 1F).

### Generation of macrophage-specific RhoA conditional knockout mice

**Fig. 2:**
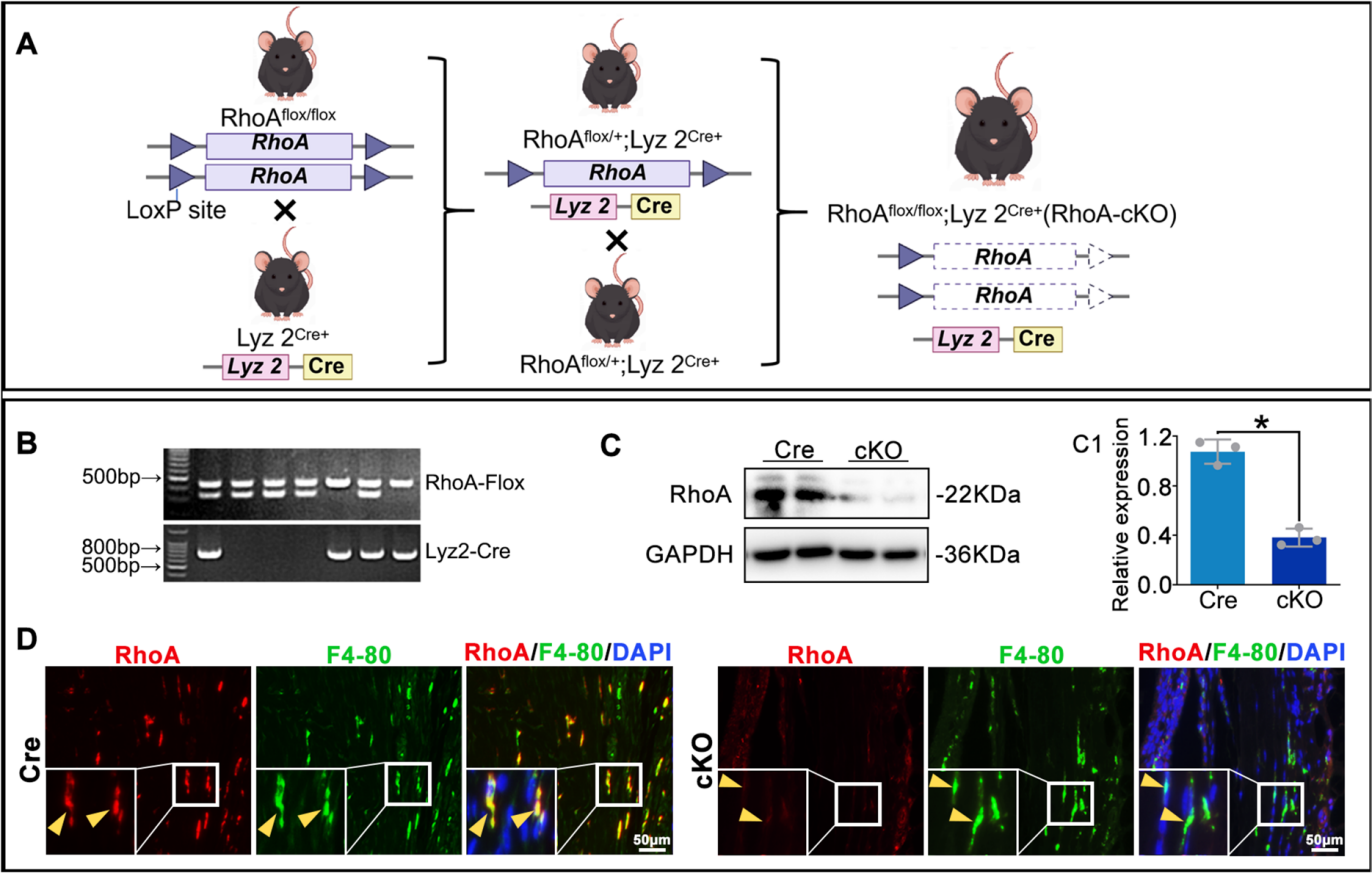
Generation and validation of macrophage-specific RhoA conditional knockout mice. **A**. Schematic of the breeding strategy. **B**. Genotyping for identification of Lyz2^Cre^; RhoA^flox/flox^ mice. **C**. WB analysis of RhoA knockout efficiency in primary macrophages. **D**. IF evaluation of RhoA expression in synovial macrophages 8 weeks post-DMM surgery. *P < 0.05

Previous studies on RhoA in OA relied heavily on pharmacological interventions, where observed effects could stem from both direct and indirect actions across multiple cell types. To specifically investigate the role of macrophage RhoA upregulation in OA, we generated macrophage-specific RhoA conditional knockout mice (Lyz2^Cre^; RhoA^flox/flox^, cKO) by crossing Lyz2^Cre^ mice with RhoA^flox/flox^ mice; littermate Lyz2^Cre^ mice served as controls (Fig. 2A, B). At 8 weeks of age, both cKO and control mice underwent OA modeling. WB and IF confirmed that RhoA was nearly undetectable in macrophages from cKO mice compared to controls (Fig. 2C, D). These results validate the successful generation of macrophage-specific RhoA cKO mice for subsequent studies.

### Macrophage-specific RhoA knockout exacerbates cartilage matrix loss in OA

**Fig. 3:**
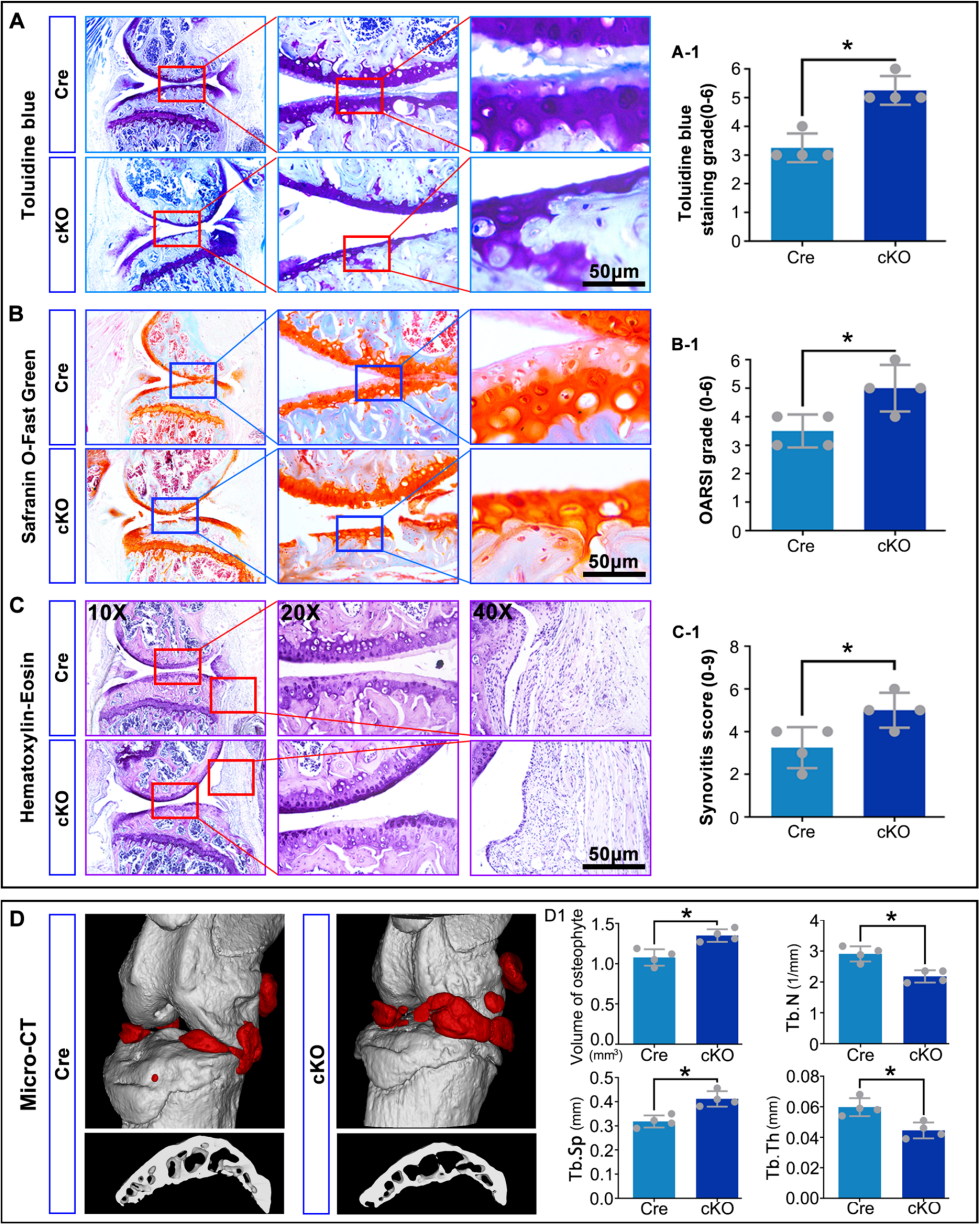
Macrophage RhoA deletion exacerbates OA cartilage matrix destruction. **A**, **B**. Cartilage matrix loss assessed by Toluidine Blue and Safranin O staining. **C**. Synovial inflammation and cartilage integrity evaluated by H&E staining. **D**. Three-dimensional reconstruction and quantitative analysis of osteophyte volume and subchondral bone parameters (Tb.N, Tb.Th, Tb.Sp) by micro-CT. *P < 0.05

At 8 weeks post-OA modeling, Toluidine Blue and Safranin O staining revealed significantly greater cartilage matrix destruction in cKO mice compared to controls (Fig. 3A, B). Hematoxylin and eosin (H&E) staining indicated increased synovial inflammation in the cKO group (Fig. 3C). Micro-CT analysis showed a marked increase in osteophyte volume, decreased trabecular number (Tb.N) and thickness (Tb.Th), and increased trabecular separation (Tb.Sp) in cKO mice (Fig. 3D). These findings suggest that deletion of macrophage RhoA promotes cartilage damage and subchondral bone loss, thereby exacerbating OA progression.

### RhoA ablation promotes M1 and suppresses M2 macrophage polarization

**Fig. 4:**
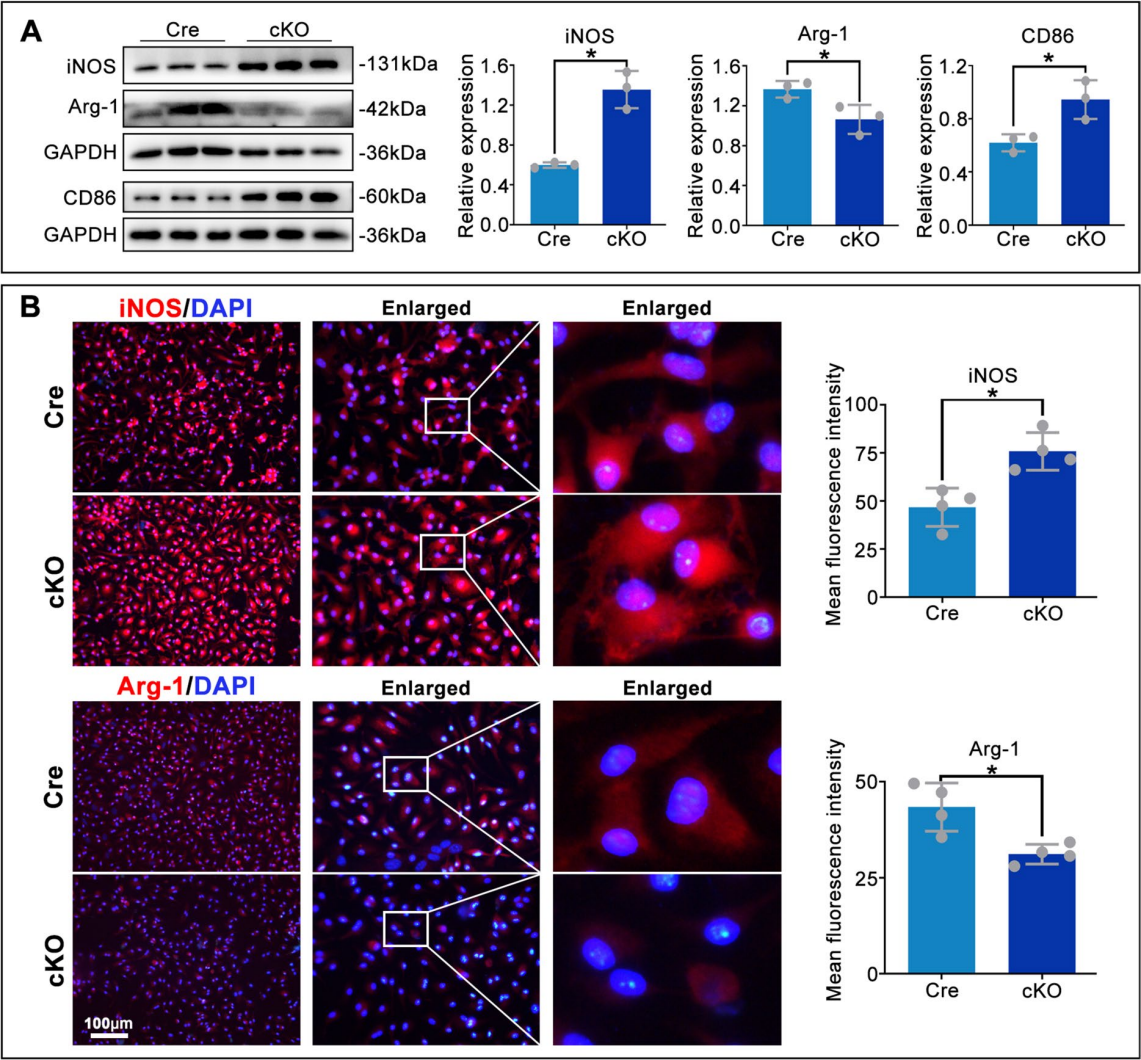
Macrophage RhoA deletion promotes pro-inflammatory polarization. **A**. Representative WB and densitometric analysis of iNOS, Arg-1, and CD86 in macrophages from Cre and cKO mice. **B**. Representative IF images and integrated fluorescence intensity of iNOS and Arg-1 in macrophages. *P < 0.05

To determine whether RhoA regulates macrophage polarization, we performed WB and IF analyses. Macrophage-specific RhoA deletion significantly increased protein levels of the M1 markers iNOS and CD86, while reducing the M2 marker Arg-1, compared to controls (Fig. 4A). IF quantification confirmed a significant increase in iNOS signal intensity and a decrease in Arg-1 in cKO macrophages (Fig. 4B). Together, these data indicate that RhoA ablation shifts macrophage polarization toward a pro-inflammatory M1 phenotype and away from an anti-inflammatory M2 phenotype.

### Conditioned medium from cKO macrophages accelerates senescence in mouse chondrocytes and human OA chondrocytes

**Fig. 5:**
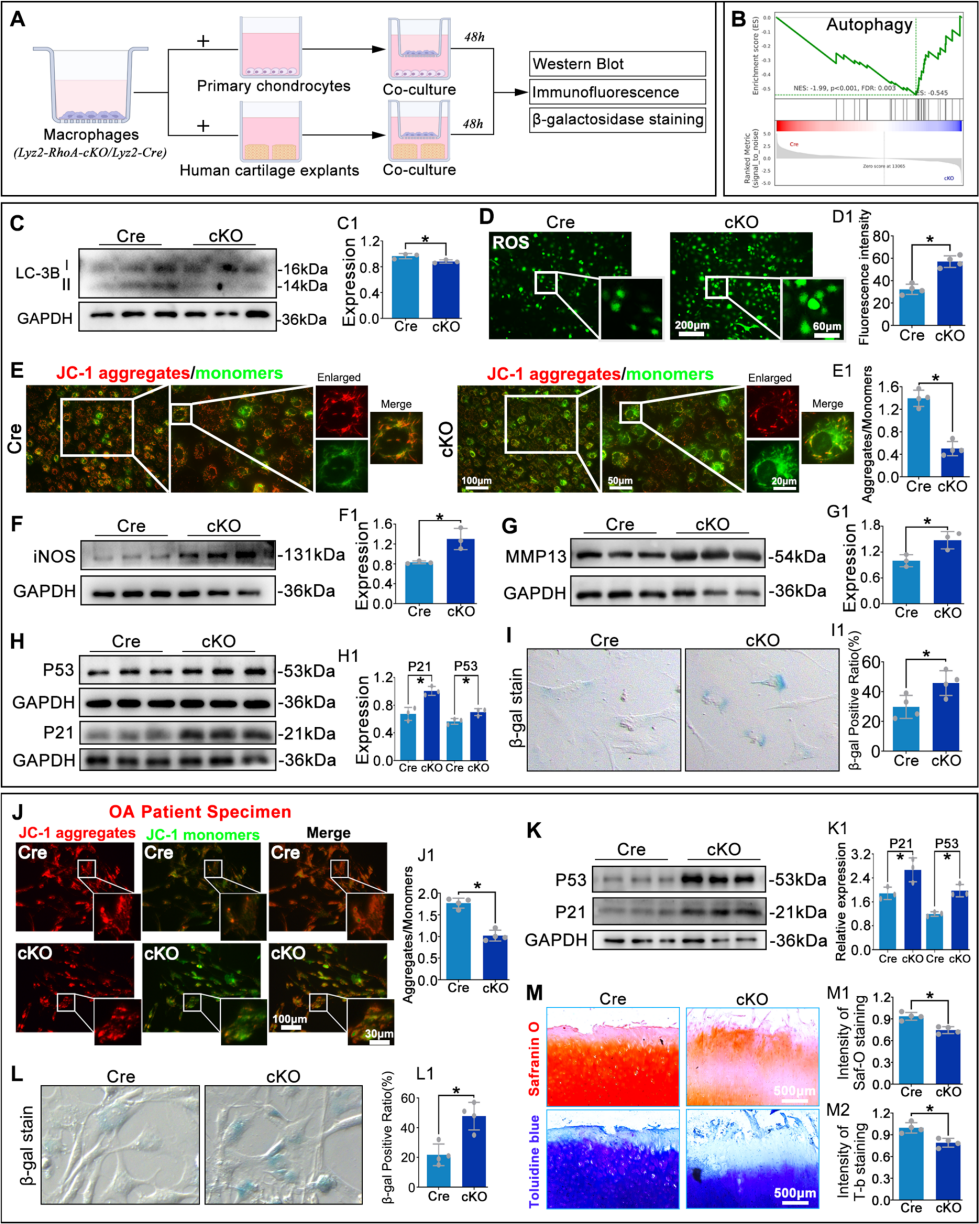
Macrophage RhoA deletion exacerbates chondrocyte senescence. **A**. Schematic of the co-culture model. **B**. GSEA showing negative enrichment of the autophagy gene set in chondrocytes from the cKO group. **C**. WB analysis of the LC3B-II/I ratio in mouse chondrocytes. **D**. IF staining for ROS using DCFH-DA. **E**. JC-1 staining for mitochondrial membrane potential in mouse chondrocytes; aggregates (red) indicate healthy potential, monomers (green) indicate depolarization. **F**–**H**. WB analysis of iNOS, MMP13, p53, and p21 in mouse chondrocytes. **I**. Quantification of SA-β-gal-positive senescent mouse chondrocytes. **J**. JC-1 staining and quantitative analysis of mitochondrial membrane potential in human OA chondrocytes. **K**,** L**. WB analysis and SA-β-gal staining for senescence markers (p53, p21) and senescent cells in human OA chondrocytes, respectively. **M**. Safranin O and Toluidine Blue staining assessing proteoglycan loss in human OA cartilage explants. *P < 0.05

To assess the impact of macrophage RhoA knockout on chondrocytes, we established a co-culture system in which Cre or cKO macrophages were incubated with either wild-type (WT) primary mouse chondrocytes or human OA cartilage explants/chondrocytes, according to the schematic design (Fig. 5A). We conducted transcriptomic profiling using RNA sequencing (RNA-seq) with co-cultured mouse chondrocytes, and Gene Set Enrichment Analysis (GSEA) revealed negative enrichment (NES = −1.99, FDR = 0.003) of an autophagy gene set in cKO group (Fig. 5B). Consistent with the GSEA findings, WB analysis showed decreased LC3-II/I ratio, indicating the autophagy were reduced in chondrocytes co-cultured with cKO macrophages compared with the control group (Fig. 5C). ROS and JC-1 staining demonstrated significantly higher reactive oxygen species (ROS) levels (Fig. 5D) and a marked reduction in healthy mitochondria (JC-1 aggregates) in chondrocytes from the cKO group (Fig. 5E). WB analysis revealed upregulation of iNOS, MMP13, p53, and p21 in chondrocytes from cKO group (Fig. 5F-H). Senescence-associated β-galactosidase (SA-β-gal) staining showed a significant increase in senescent chondrocytes in the cKO group (Fig. 5I). Similarly, the human OA chondrocytes co-cultured with cKO macrophages showed reduced mitochondrial membrane potential (lower JC-1 red/green ratio), elevated senescence markers (p53, p21, SA-β-gal), and accelerated cartilage matrix loss compared with control group, as shown by Safranin O and Toluidine Blue staining (Fig. 5J-M). These data demonstrate that macrophage RhoA deletion enhances chondrocyte autophagy suppression, ROS production, and mitochondrial injury, thereby accelerating chondrocyte senescence.

### IL-17C mediates the pro-senescent effect of macrophage RhoA deletion on chondrocytes

**Fig. 6:**
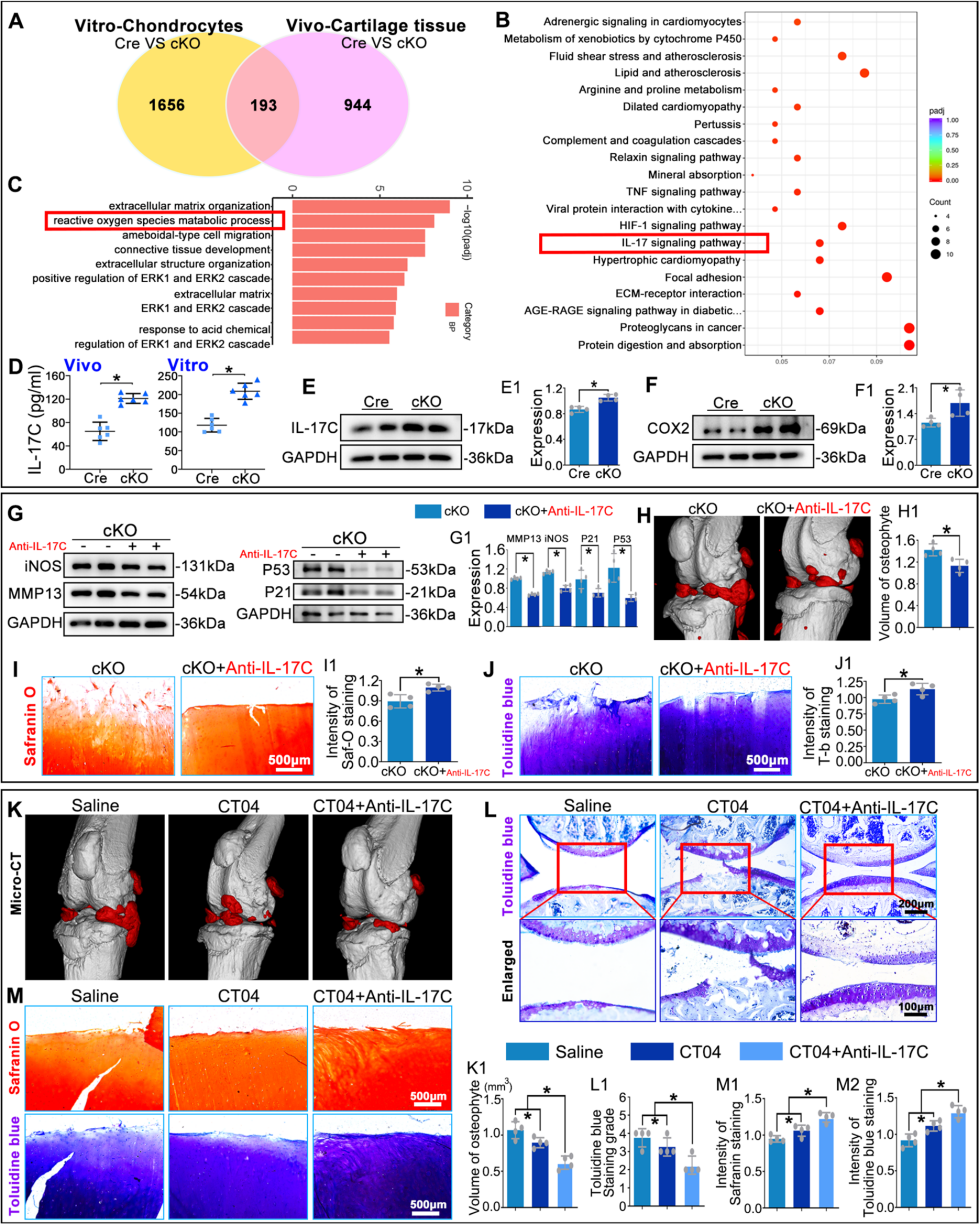
IL-17C mediates macrophage RhoA-induced chondrocyte senescence. **A**-**C**. Integrated transcriptome analysis of mouse OA cartilage (Red) and co-cultured chondrocytes (Yellow). **D**. IL-17C secretion in joint fluid and macrophage supernatants measured by ELISA. **E**. WB analysis of IL-17C in macrophages. **F**. WB analysis of COX2 in co-cultured chondrocytes. **G**. WB analysis of iNOS, MMP13, p53, and p21 in co-cultured chondrocytes after Anti-IL-17C treatment. **H**. Micro-CT 3D reconstruction and quantification of osteophyte volume in OA mice. **I**,** J**. Safranin O and Toluidine Blue staining assessing cartilage matrix loss in human OA explants. **K**. Micro-CT analysis of osteophyte volume in OA mice. **L**. Toluidine Blue staining of cartilage matrix in OA mice. **M**. Safranin O and Toluidine Blue staining of matrix loss in human OA explants. *P < 0.05

To identify mechanisms by which macrophage RhoA influences chondrocytes, we performed RNA-seq on cartilage from OA mice (Lyz-Cre vs. Lyz-RhoA-cKO) and on mouse chondrocytes co-cultured with macrophages. Intersection analysis identified 193 common differentially expressed genes (Fig. 6A). KEGG enrichment analysis revealed significant enrichment of the IL-17 signaling pathway relevant to OA (Fig. 6B), and GO analysis showed that the 'reactive oxygen species metabolic process' was among the significantly enriched biological processes (Fig. 6C). ELISA of joint fluid from OA mice and macrophages supernatants, along with WB analysis of macrophages, showed that the secretion and expression of IL-17C, but not IL-17A or IL-17B, were significantly upregulated in the cKO group (Fig. 6D, E. S. Figure 1A, B). Furthermore, COX2, a key effector of IL-17 signaling, was elevated in chondrocytes co-cultured with cKO macrophages compared to the control group (Fig. 6F). These results suggest that macrophage RhoA deletion activates the IL-17 signaling pathway in chondrocytes via IL-17C secretion, influencing ROS metabolism and thus promoting OA.

To further validate IL-17C's role, the IL-17C-neutralizing antibody was added into the co-culture system in which cKO macrophages were incubated with WT primary mouse chondrocytes or human OA cartilage explants,(100 µM, S. Figure 1C), and was administered via intra-articular injection to cKO mice (10 mg/kg). WB showed reduced expression of iNOS, MMP13, p53, and p21 in chondrocytes from the cKO + Anti-IL-17C group compared to the cKO alone (Fig. 6G). Micro-CT analysis revealed that Anti-IL-17C injection significantly reduced osteophyte formation in cKO mice (Fig. 6H). Safranin O and Toluidine Blue staining demonstrated that Anti-IL-17C mitigated cartilage matrix destruction induced by cKO macrophages in human OA explants (Fig. 6I, J). These results indicate that macrophage RhoA deletion accelerates cellular senescence and OA progression via enhanced IL-17 signaling.

Given that macrophage RhoA deletion drives OA through excessive IL-17C secretion, we explored combining an IL-17C-neutralizing antibody with RhoA inhibition. CT04 (a RhoA activity inhibitor) alone or CT04 + Anti-IL-17C was added into the co-culture system in which Cre macrophages were incubated with human OA cartilage explants, and was administered via intra-articular injection to Cre mice. Micro-CT showed a significant reduction in osteophyte volume in the CT04 + Anti-IL-17C group compared to CT04 alone (Fig. 6K). Toluidine Blue staining revealed less cartilage matrix damage in mice treated with the combination therapy (Fig. 6L). Similarly, Safranin O and Toluidine Blue staining showed the matrix loss in explants was attenuated in the combination group (Fig. 6M). These results indicate that IL-17C neutralization effectively blocks the detrimental effects of macrophage RhoA deletion and enhances the therapeutic efficacy of RhoA inhibition in OA.

### IL-17C promotes chondrocyte senescence by activating the PI3K/AKT/mTOR axis

**Fig. 7:**
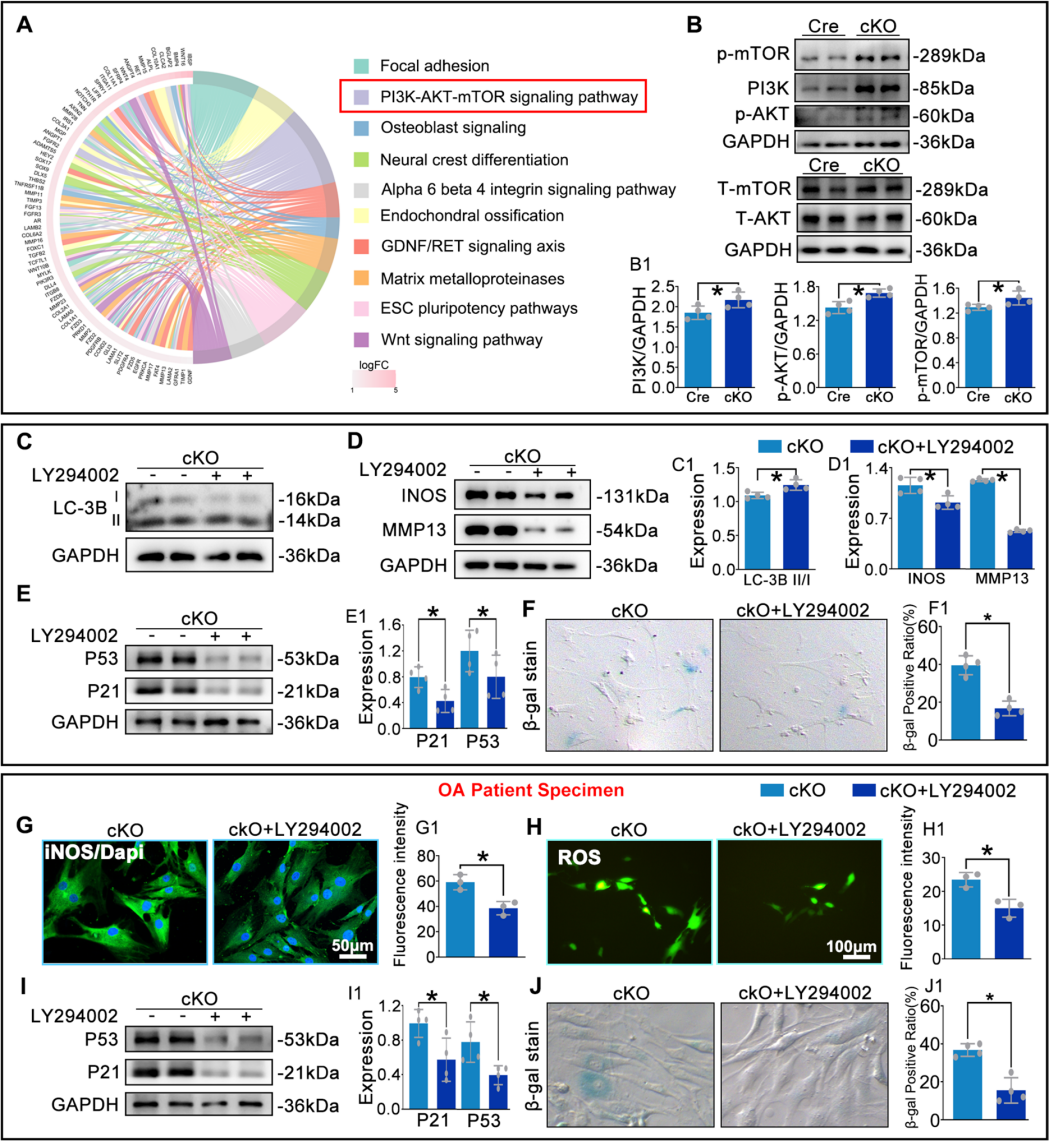
IL-17C accelerates chondrocyte senescence via PI3K/AKT/mTOR activation. **A**. WikiPathways enrichment analysis of RNA-seq data from chondrocytes co-cultured with macrophages. **B**. WB analysis of PI3K, AKT, mTOR, p-AKT, and p-mTOR in co-cultured chondrocytes. **C**-**F**. WB and SA-β-gal assays quantifying LC3B, iNOS, MMP13, p53, p21, and senescent mouse chondrocytes. **G**,** H**. IF analysis of iNOS and ROS in human OA chondrocytes. **I**,** J**. WB and SA-β-gal assays quantifying p53, p21, and senescent human OA chondrocytes. *P < 0.05

RNA-seq of chondrocytes revealed significant enrichment of the PI3K-AKT-mTOR signaling pathway among upregulated WikiPathways (Fig. 7A). Given prior reports indicate that (i) IL-17 can activate PI3K-AKT-mTOR signaling(Faust et al. 2020; Zhou et al. 2020b), (ii) mTOR activation suppresses autophagy(Li et al. 2025; Qiu et al. 2025), and (iii) autophagy inhibition promotes senescence(Chen et al. 2025; Feng et al. 2024), we hypothesized that IL-17C links these events to accelerate cartilage senescence.

WB analysis confirmed significantly increased levels of PI3K, phosphorylated AKT (p-AKT), and phosphorylated mTOR (p-mTOR) in chondrocytes co-cultured with cKO macrophages compared to the control group (Fig. 7B). Chondrocytes co-cultured with cKO macrophages showed suppressed autophagy (lower LC3B-II/I ratio), elevated inflammation (iNOS/MMP13) and senescence (increased p53/p21 and SA-β-gal-positive cells), which were markedly attenuated by the treatment with a PI3K inhibitor (Fig. 7C-F). In co-cultured human OA chondrocytes, PI3K inhibition similarly reduced iNOS expression, ROS levels, p53/p21 protein abundance, and the proportion of SA-β-gal-positive cells compared to the cKO group (Fig. 7G-J). Collectively, these results indicate that macrophage RhoA deletion promotes chondrocyte senescence via the IL-17C-PI3K-AKT-mTOR signaling axis.

### RhoA regulates IL-17C expression in macrophages via the LATS/YAP/CCN2 axis

**Fig. 8:**
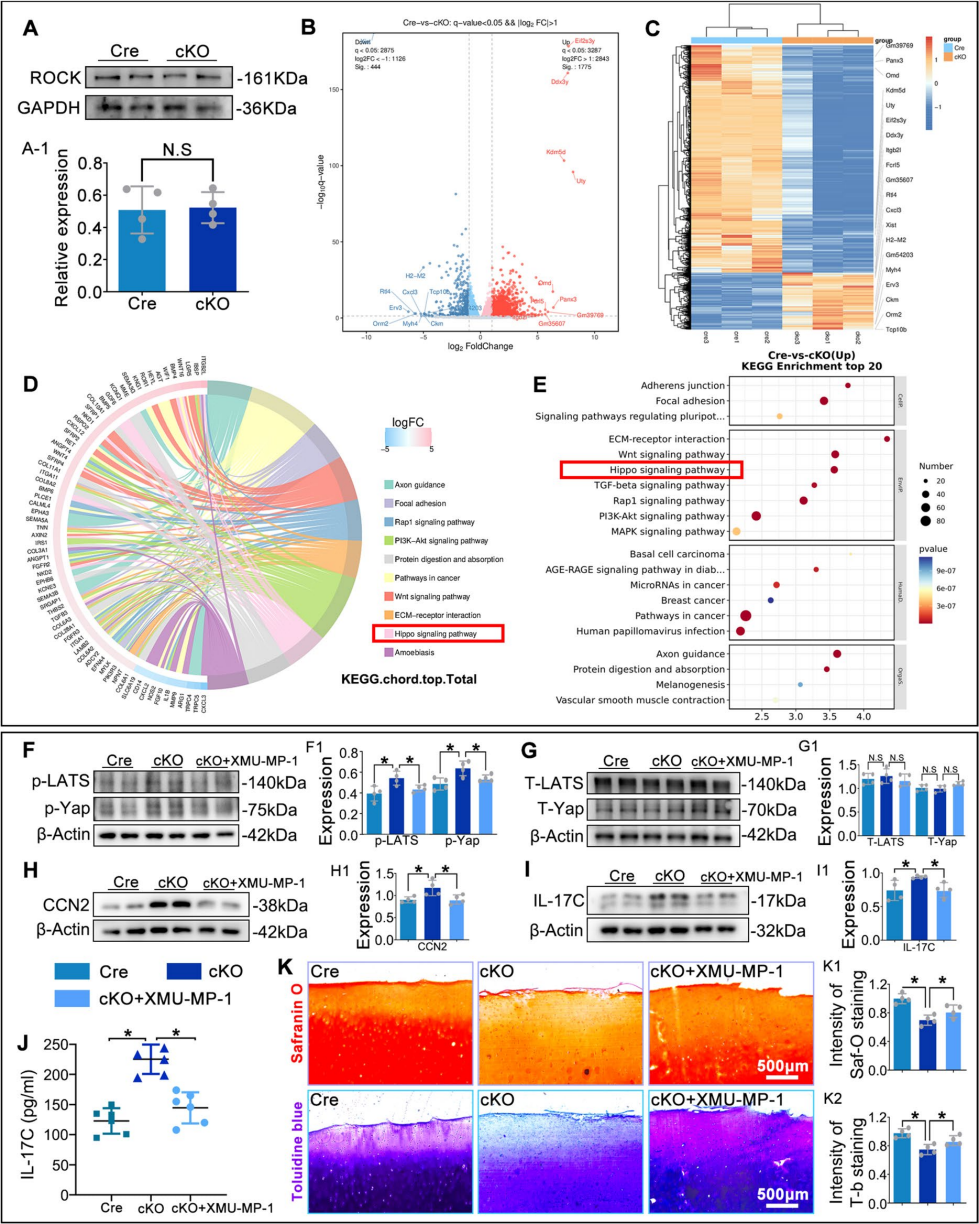
Macrophage RhoA regulates IL-17C secretion via the LATS/YAP/CCN2 signaling axis. **A**. WB analysis of ROCK protein levels in macrophages. **B**-**F**. RNA-seq analysis of Cre vs. cKO macrophages. **B**. Volcano plot of differentially expressed genes (DEGs). **C**. Heatmap of DEGs across samples. **D**,** E**. KEGG enrichment analysis of DEGs. **F**-**I**. WB analysis of LATS, YAP, their phosphorylated forms, CCN2 and IL-17C in macrophages. **J**. ELISA of IL-17C in macrophage supernatants. **K**. Safranin O and Toluidine Blue staining assessing matrix loss in human OA cartilage explants. *P < 0.05. N.S.: not significant

To elucidate how macrophage RhoA regulates OA progression, we first examined its canonical downstream effector ROCK. WB analysis showed that macrophage-specific RhoA deletion did not significantly alter ROCK expression in macrophages (Fig. 8A), suggesting that RhoA acts independently of ROCK in this context. RNA-seq of primary macrophages from control and cKO mice revealed that RhoA deletion upregulated 1775 genes and downregulated 444 genes (Fig. 8B-D). KEGG analysis of differentially expressed genes identified the top 20 enriched pathways, with literature review highlighting a correlation between the Hippo signaling pathway, OA progression, and the IL-17 pathway (Fig. 8E). WB analysis showed a significant upregulation of phosphorylated LATS (p-LATS), phosphorylated YAP (p-YAP), together with their downstream target CCN2, indicating Hippo pathway activation in RhoA-deleted macrophages. Treatment with the Hippo pathway inhibitor XMU-MP-1 (400 µM) (Li et al. 2024) significantly reduced IL-17C protein expression and secretion in cKO macrophages, as shown by WB analysis and ELISA (Fig. 8F-J). Co-culture of human OA chondrocyte explants with XMU-MP-1-treated cKO macrophages resulted in reduced matrix loss compared to untreated cKO macrophages (Fig. 8K). These results suggest that RhoA deletion in macrophages promotes IL-17C secretion and exacerbates OA progression via activation of the LATS/YAP/CCN2 signaling axis.

## Discussion

This study reveals a novel and critical role for macrophage-specific RhoA signaling in alleviating OA progression, which is distinct from its reported roles in chondrocytes or endothelial cells in OA pathogenesis (He et al. 2024; Jiang et al. 2023). While previous literature has firmly established RhoA/ROCK as a promoter of cartilage catabolism and subchondral bone pathology (Yang et al. 2023; Zhu et al. 2013), our findings paradoxically demonstrate that RhoA cKO in the synovial macrophage compartment exacerbates OA disease. This cell-type-specific function underscores the complex, context-dependent nature of RhoA signaling in the joint and highlights the limitations of systemic pharmacological inhibition.

As key players in the innate immune response within the joint cavity, synovial macrophages respond to danger-associated molecular patterns, such as necrotic cell debris and cartilage fragments, by releasing cytokines that activate chondrocyte receptor signalling, thereby contributing to OA exacerbation (Chen et al. 2022; Yin et al. 2024; Zhao et al. 2023). This suggests three potential strategies for targeting the detrimental effects of synovial macrophages: (1) blocking the source by elucidating the pathological mechanisms and signalling alterations in synovial macrophages under OA conditions (Blom et al. 2007; Hamasaki et al. 2020; Zhang et al. 2018); (2) targeting the action pathways by identifying and neutralising secreted pathogenic factors (Liu et al. 2023; Mikulkova et al. 2024); (3) intervening at downstream targets by understanding changes in chondrocyte receptors and signalling pathways to inhibit downstream cascades (Ebata et al. 2021). Present key finding is the identification of IL-17C as the primary effector links the cross-talk of RhoA-deficient macrophages with chondrocyte senescence. The IL-17 family, particularly IL-17A, has been implicated in OA (Faust et al. 2020); however, the role of IL-17C is still unknow. The evidences of current study demonstrate that RhoA acts as a transcriptional brake on IL-17C in macrophages, and its deletion unleashes a cascade of events: heightened IL-17C secretion, activation of the PI3K/AKT/mTOR axis in chondrocytes, suppression of protective autophagy, and ultimately, the induction of a senescent, catabolic chondrocyte phenotype. This macrophage-chondrocyte crosstalk via the RhoA/IL-17C axis represents a significant conceptual advance in understanding OA pathogenesis.

Another interesting finding of this study is that RhoA deletion in macrophages activated YAP/CCN2 signaling without altering ROCK expression reveals a non-canonical pathway for RhoA in immune regulation. Previous studies have indicated that RhoA, upon oxidation by mitochondrial superoxide, activates YAP/TAZ, leading to liver damage in injury models (Kwon et al. 2024). It has been reported that CCN2 enhances IL-17 synthesis by disrupting the inhibitory effect of miR655 on IL-17 (Zhang et al. 2024). This shifts the paradigm away from the classic RhoA-ROCK cytoskeletal axis and towards the Hippo/YAP pathway as a key regulator of inflammatory cytokine production in OA macrophages. Which suggests that therapeutic strategies targeting RhoA for OA treatment must be highly cell-type-specific, as global inhibition could simultaneously block protective pathways in macrophages and pathogenic pathways in other joint cells. For example, Allen et al. demonstrated that opposing RhoA actions in spinal neurons versus astrocytes restrict regeneration; only neuron-specific RhoA inhibition or blockade of its astrocyte-proliferative effectors fully unleashes the pathway’s repair potential (Stern et al. 2021). Second, this study indicates IL-17C as a promising and more tractable therapeutic target. The synergistic benefit observed when combining a RhoA activator (CT04) with an IL-17C-neutralizing antibody provides a compelling preclinical rationale for a multi-targeted approach to mitigate macrophage-driven joint destruction.

In conclusion, our results demonstrate a previously unknown RhoA/YAP/IL-17C signaling axis in synovial macrophages that serves as a critical pathway against OA progression. By IL-17C paracrine, macrophage RhoA plays important role in maintaining chondrocyte homeostasis and cartilage integrity. This study not only redefines the function of RhoA in OA but also opens new avenues for immunomodulatory therapies aimed at disrupting the pathogenic crosstalk between synovial inflammation and cartilage senescence.

## Supplementary Information

Below is the link to the electronic supplementary material.

Supplementary file1 (DOCX 142 KB)

Supplementary file2 (DOCX 22 KB)

Supplementary file3 (PDF 8791 KB)

## Data Availability

The data from this study are available from the author for correspondence upon reasonable request.
