# Supplementary material for: Synovial macrophage rhoa protects against osteoarthritis by suppressing YAP/IL-17C mediated chondrocyte senescence
Source: Cell Biol Toxicol. 2026 Jan 31;42(1):30. doi: 10.1007/s10565-026-10151-w (PMC12894126; doi:10.1007/s10565-026-10151-w)
Supplement: Supplementary file 1 — Supplementary file1 (DOCX 142 KB) [file 10565_2026_10151_MOESM1_ESM.docx]

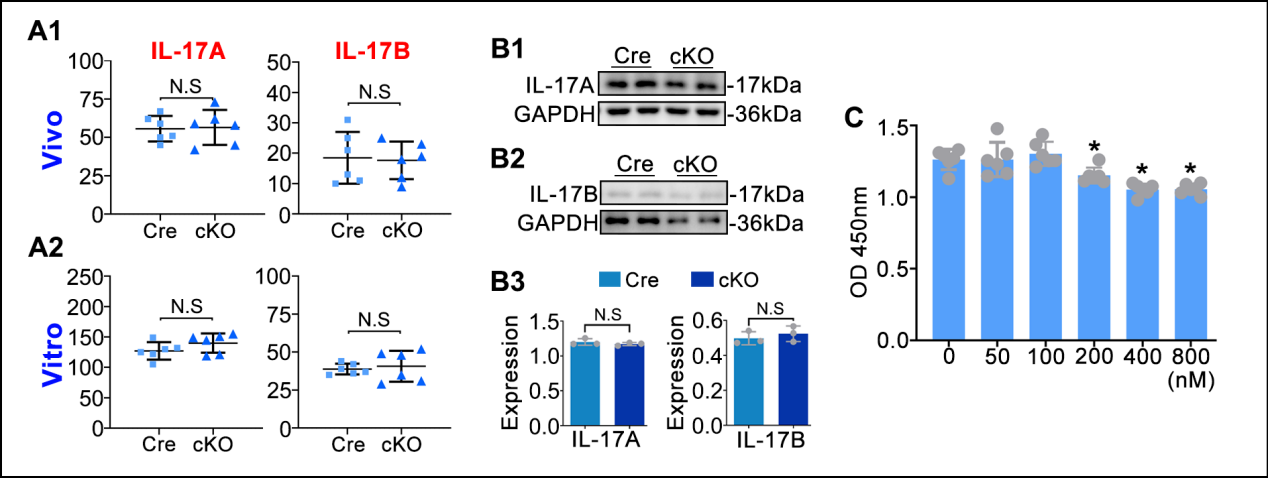


**Figure S1: A.** ELISA measuring IL-17A and IL-17B expression levels in joint fluid of OA mice (Lyz-Cre/Lyz-RhoA-cKO) and macrophage supernatants (Lyz-Cre/Lyz-RhoA-cKO). **B.** WB assessment of IL-17A and IL-17B protein expression levels in primary macrophages (Lyz-Cre/Lyz-RhoA-cKO). **C.** CCK8 detects the toxic effect of Anti-IL-17C on macrophages. *P < 0.05, N.S: No statistical difference.
