# Supplementary material for: Synovial macrophage rhoa protects against osteoarthritis by suppressing YAP/IL-17C mediated chondrocyte senescence
Source: Cell Biol Toxicol. 2026 Jan 31;42(1):30. doi: 10.1007/s10565-026-10151-w (PMC12894126; doi:10.1007/s10565-026-10151-w)
Supplement: Supplementary file 2 — Supplementary file2 (DOCX 22 KB) [file 10565_2026_10151_MOESM2_ESM.docx]

| **Antibody** | **Source species** | **Dilution** | **Company** |
| --- | --- | --- | --- |
| Phospho-AKT | Rabbit | 1:1000 (WB) | Abclonal, CHN, Cat#AP1208 |
| AKT | Rabbit | 1:1000 (WB) | Abclonal, CHN, Cat#AP18675 |
| BAX | Rabbit | 1:1000 (WB) | Abcam, UK, Cat#ab39012 |
| BCL2 | Mouse | 1:1000 (WB) | Proteintech, USA, Cat#12789-1-AP |
| CCN2/CTGF | Rabbit | 1:1000 (WB) | Abclonal, CHN, Cat#A11067 |
| COX2 | Rabbit | 1:1000 (WB) | Abclonal, CHN, Cat#A25901PM |
| F4/80 | Rabbit | 1:100 (IF) | Abcam, UK, Cat#ab300422 |
| GAPDH | Rabbit | 1:2000 (WB) | Abcam, UK, Cat#ab8245 |
| IL-17A | Rabbit | 1:1000 (WB) | Abclonal, CHN, Cat#A12454 |
| IL-17B | Rabbit | 1:1000 (WB) | Affinity, USA, Cat#DF8979 |
| IL-17C | Rabbit | 1:1000 (WB) | Abclonal,CHN,Cat#A10587 |
| IL-17A | Mouse | ELISA | Boster, CHN, Cat#EK0431 |
| IL-17B | Mouse | ELISA | Abcam, UK, Cat#ab100703 |
| IL-17C | Mouse | ELISA | CLOUD-CLONE CORP, CHN, SED347Mu |
| iNOS | Rabbit | 1:1000 (WB),  1:400 (IF) | Abcam, UK, Cat#ab178945 |
| Phospho-LAST1/2 | Rabbit | 1:1000 (WB) | Abclonal, CHN, Cat#AP1517 |
| LAST | Rabbit | 1:1000 (WB) | Abcam, UK, Cat#ab70561 |
| LC-3B | Rabbit | 1:1000 (WB) | Abcam, UK, Cat#ab48394 |
| MMP13 | Rabbit | 1:1000 (WB) | Abcam, UK, Cat#ab39012 |
| Phospho-mTOR | Rabbit | 1:1000 (WB) | Abclonal, CHN, Cat#AP0490 |
| mTOR | Rabbit | 1:1000 (WB) | Abclonal, CHN, Cat#A11355 |
| P53 | Rabbit | 1:1000 (WB) | Affinity, USA, Cat#BF8013 |
| P21 | Rabbit | 1:1000 (WB) | Zenbio, CHN, Cat#R381102 |
| RhoA | Mouse | 1:1000 (WB), 1:400 (IF), 1:100 (IHC) | Santa Cruz, Cat# sc-418 |
| Phospho-YAP | Rabbit | 1:1000 (WB) | Abclonal, CHN, Cat#AP1436 |
| YAP | Rabbit | 1:1000 (WB) | Abclonal, CHN, Cat#AP22650 |
| HRP-conjugated secondary antibody | Rabbit | 1:2000 (WB) | Molecular Probes, USA, Cat#65-6120 |
| HRP-conjugated secondary antibody | Mouse | 1:2000 (WB) | Invitrogen, USA,  Cat#SA5-10317 |
| Goat anti-rabbit Alexa Fluor 568 | Goat | 1:400 (IF) | Invitrogen, USA,  Cat#A-11011 |
| Goat anti-rabbit Alexa Fluor 488 | Goat | 1:400 (IF) | Invitrogen, USA,  Cat#A-11008 |
| Goat anti-mouse Alexa Fluor 488 | Goat | 1:400 (IF) | Invitrogen, USA,  Cat#A-21121 |
| Goat anti-mouse Alexa Fluor 568 | Goat | 1:400 (IF) | Invitrogen, USA,  Cat#A-11004 |
| Universal two-step test | Mouse/Rabbit | Undiluted  (IHC) | ZSGB-BIO, CHN,  Cat#PV-9000‌‌ |
| Anti-IL-17C | / | / | Selleck, USA, A2450 |
| LY294002 | / | / | MCE, USA, HY-10108 |
| XMU-MP-1 | / | / | MCE, USA, HY-100526 |
| ROS | / | / | Beyotime, CHN, Cat#S0033S |
| JC-1 | / | / | Beyotime, CHN, Cat#C2006 |

**Table S1.** The list of antibodies used in present study.

Note: **WB** means western blot, **IF** means immunofluorescence, **IHC** means immunohistochemistry, **ELISA** means Enzyme-Linked Immunosorbent Assay.
