# Supplementary material for: Synovial macrophage rhoa protects against osteoarthritis by suppressing YAP/IL-17C mediated chondrocyte senescence
Source: Cell Biol Toxicol. 2026 Jan 31;42(1):30. doi: 10.1007/s10565-026-10151-w (PMC12894126; doi:10.1007/s10565-026-10151-w)
Supplement: Supplementary file 3 — Supplementary file3 (PDF 8791 KB) [file 10565_2026_10151_MOESM3_ESM.pdf]

96% Unique

Total 33743 chars, 4778 words, 344 unique sentence(s).

Instant Essay Writing by AI - ***Instant essay writing service by AI. You can buy essays, dissertations, courseworks, thesis papers. We will write your English paper, do your assignment and homework. 100% artificial intelligence custom writing, no plagiarism. Papers ready in 5 minutes! Prices from $5/page.***

Results	Query	Domains (original links)
Unique	Abstract Objective: The GTPase RhoA is known as a regulator involved in cartilage degeneration and	-
Unique	However, its specific role in synovial macrophages, the key immune cells of OA related tissues,	-
Unique	Methods: Herein, the RhoA expression in human and mouse OA synovium was analyzed	-
Unique	A macrophage-specific RhoA conditional knockout (cKO) mouse model was generated	-
Unique	Histological staining, OARSI scoring, and micro-CT were used to assess cartilage damage, while Western blot,	-
Unique	Transcriptome sequencing and validation of signaling pathways were conducted using tissues and cells from patients	-
Unique	Results: The collected results indicate that RhoA expression was significantly upregulated in synovial macrophages from	-
Unique	Contrary to its reported role in chondrocytes or endothelial cells, macrophage-specific RhoA deletion exacerbated OA,	-
Unique	RhoA-deficient macrophages exhibited a pro-inflammatory M1 polarization and secreted high levels of IL-17C	-
Unique	This cytokine was necessary and sufficient to induce chondrocyte senescence, as evidenced by increased p53/p21,	-
Unique	Mechanistically, RhoA ablation in macrophages activated the Hippo pathway effectors YAP/CCN2, leading to IL-17C transcription,	-
Unique	Conclusion: In conclusion, present study reveals a previously unrecognized, protective role for macrophage RhoA in	-
Unique	It functions as a critical brake on a novel YAP-IL-17C axis, thereby preserving chondrocyte	-
Unique	This study redefines RhoA's role in joint homeostasis and nominates IL-17C as a potential therapeutic	-
Unique	Keywords: Osteoarthritis; RhoA; Synovial Macrophage; Chondrocyte Senescence; YAP/IL-17C Introduction Osteoarthritis (OA) is a highly prevalent	-
Unique	2023; Hunter and Bierma-Zeinstra 2019; Roelofs and De Bari 2024)	-

Unique	OA is well known as its progressive synovial inflammation, cartilage deterioration, and subchondral bone remodeling,	-
Unique	2024; Kloppenburg 2023; Xu et al	-
Unique	2023)	-
Unique	Among the various signaling pathways implicated in OA, the Rho GTPase RhoA has garnered increasing	-
Found	2019; Novakofski et al	sciencedirect.com pmc.ncbi.nlm.nih.gov
Unique	2009)	-
Unique	Accumulating evidence positions RhoA as a central regulator of cytoskeletal dynamics, cell differentiation, and inflammatory	-
Unique	Studies have documented its pathogenic role in promoting chondrocyte hypertrophy, cartilage matrix degradation, and vascular	-
Unique	2022; Wang and Beier 2005; Wang et al	-
Found	2004; Zhang et al	researchgate.net agupubs.onlinelibrary.wiley.com jstor.org ajol.info
Unique	2025)	-
Unique	However, the existing researches primarily focused on RhoA functions in chondrocytes and endothelial cells(He et	-
Unique	2024; Jiang et al	-
Unique	2023)	-
Unique	The role of RhoA in synovial macrophages, key sentinels and amplifiers of joint inflammation, has	-
Unique	Given that macrophage polarization critically influences OA progression(Cai et al	-
Unique	2025; Jiang et al	-
Found	2025; Wang et al	frontiersin.org
Unique	2025), we hypothesized that RhoA signaling within these immune cells could be a pivotal regulator	-
Unique	In this study, we systematically investigated the expression and functional significance of RhoA in synovial	-
Unique	Combined with clinical OA samples, experimental OA model in wild type mice and macrophage-specific conditional	-
Unique	Mechanically, macrophages RhoA modulates IL-17C secretion through the non-canonical Hippo/YAP signaling pathway, and the IL-17C	-
Unique	Present study not only broaden the understanding of RhoA in OA but also reveal a	-
Unique	Materials and methods Experimental animals and human OA samples Animals：Male C57BL/6 mice (8 weeks, 20–22	-
Unique	OA was induced by destabilization of the medial meniscus (DMM) at 8 weeks of age	-

Unique	For primary chondrocyte isolation, 3-day-old neonatal pups were euthanized by decapitation	-
Unique	All procedures were approved by the Institutional Animal Care and Use Committee of Southern Medical	-
Unique	SMU-L2018158)	-
Unique	Transgenic mice： Lyz2-Cre mice (Sangon Biotech, Suzhou, China) were backcrossed to C57BL/6 for ≥ 6 generations	-
Unique	Cre-negative RhoAflox/flox littermates served as controls	-
Unique	Genotyping was performed following the protocols described in our previous publication(Xu et al	-
Unique	2021)	-
Unique	Human tissues： Synovial samples were obtained from patients with end-stage OA (KL grade 1–4; mean age	-
Unique	The study protocol was approved by the Institutional Review Board (approval no	-
Unique	TY-ZKY2024-141-01), and written informed consent was obtained from all participants	-
Unique	Primary cell and OA cartilage culture Primary chondrocytes were isolated from 3-day-old C57BL/6 pups by	-
Unique	Bone marrow–derived macrophages (BMDMs) were generated by flushing femora and tibiae from 8-week-old mice and	-
Unique	2021)	-
Unique	Human OA cartilage was obtained from total-knee-replacement patients within 2 h of surgery, minced into	-
Unique	2023)	-
Unique	All human tissues were collected under approval TY-ZKY2024-141-01 with written informed consent	-
Unique	Animal surgery and drug administration OA was induced by destabilisation of the medial meniscus (DMM)	-
Unique	Under sterile conditions and continuous isoflurane anaesthesia (2 % in O_2, 1 L/min), the medial	-
Unique	Sham mice underwent identical arthrotomy without ligament transection(Xu et al	-
Unique	2023)	-
Unique	Post-operative analgesia (Carprofen 5 mg/kg s.c	-
Unique	every 12 h for 48 h) and prophylactic antibiotics (enrofloxacin 5 mg/kg s.c	-
Unique	once daily for 3 days) were administered	-
Unique	Beginning 7 days after surgery, mice received weekly intra-articular injections (30-gauge needle via the patellar	-
Unique	) for 7 consecutive weeks: (1) DMM CT04: CT04 (1 μg/μL, 10 μL(Zhang et al	-
Unique	2012)) in 0.9 % NaCl, pH 7.4	-

Unique	(2) DMM CT04 Anti-IL-17C: CT04 (2 μg/μL, 5 μL) plus Anti-IL-17C (20 mg/kg, 5 μL	-
Unique	2020b))	-
Unique	(3) DMM vehicle (Control group): equal volume of sterile 0.9 % NaCl	-
Unique	Stock solutions were sterile-filtered (0.22 μm), aliquoted, and stored at −80 °C	-
Unique	Aliquots were thawed at 4 °C immediately before use, vortexed for 5 s, centrifuged 1	-
Unique	Micro-computed tomography The mouse knee tissues were preserved in a 4% paraformaldehyde solution for 72	-
Unique	The analysis assessed several parameters, including the osteophyte count, trabecular separation (Tb	-
Unique	Sp, mm), trabecular thickness (Tb.Th, mm), and trabecular number (Tb	-
Unique	N, 1/mm)	-
Unique	These parameters were quantified using the accompanying software for the micro-CT system (ZKKS-MicroCT4.1, China)	-
Unique	Histological staining Knee joints were fixed in 4 % paraformaldehyde for 24 h at 4	-
Unique	Safranin O-fast green (0.1 % Safranin O, 5 min; 0.2 % Fast Green, 5 min)	-
Unique	Cartilage degeneration was scored according to OARSI 2019 guidelines (0–6 scale) by two blinded observers	-
Unique	Synovitis was graded on H&E-stained sections (0–9 scale)	-
Unique	For immunohistochemistry, sections were deparaffinized, rehydrated, subjected to heat-mediated antigen retrieval (citrate buffer, pH 6.0,	-
Unique	Primary antibodies (dilutions in Table S1) were incubated overnight at 4 °C, followed by HRP-conjugated	-
Unique	Sections were counterstained with haematoxylin, dehydrated and mounted	-
Unique	For immunofluorescence, antigen retrieval and blocking (5 % normal goat serum, 1 h) were performed	-
Unique	Primary antibodies were incubated overnight at 4 °C, followed by Alexa Fluor-conjugated secondary antibodies (488	-
Unique	Nuclei were stained with DAPI (1 μg/mL, 5 min)	-
Unique	Images were captured using a Nikon A1 confocal microscope at identical settings	-
Unique	The list of antibodies used is provided in the Supplementary Materials Table 1	-
Unique	RNA sequencing Total RNA was extracted using Trizol Reagent (Invitrogen Life Technologies), with concentration, quality,	-
Unique	Three micrograms of RNA were used for library preparation	-
Unique	mRNA was isolated from total RNA using poly-T oligo-attached magnetic beads, followed by fragmentation using	-
Unique	First-strand cDNA was synthesized with random primers and Super-Script II, and second-strand synthesis was performed	-

Unique	Exonuclease/polymerase activities converted remaining overhangs into blunt ends, and the enzymes were removed	-
Unique	Following adenylation of the 3′ ends, Illumina PE adapter oligonucleotides were ligated for hybridization preparation	-
Unique	To select cDNA fragments of 400-500 bp, the library was purified using the AMPure XP	-
Unique	Adapter-ligated DNA fragments were enriched through 15 cycles of PCR using Illumina PCR Primer Cocktail	-
Unique	The products were purified (AMPure XP system) and quantified using the Agilent high-sensitivity DNA assay	-
Unique	The sequencing library was processed on the NovaSeq 6000 platform (Illumina) at Shanghai Personal Biotechnology	-
Unique	Ltd	-
Unique	Transcriptome analysis followed the manufacturer's protocol	-
Unique	Single-cell RNA sequencing data processing The single-cell RNA sequencing dataset GSE133449(Sun et al	-
Unique	2020) (https://doi.org/ 10.1136/annrheumdis-2019-215926) was processed using the Seurat R package	-
Unique	These data were originated from the patients with OA (n = 4)	-
Unique	Initial quality control steps included filtering cells with high mitochondrial gene content (20%) and	-
Unique	Normalization and scaling of the data were performed, followed by the identification of highly variable	-
Unique	A differential expression analysis was performed on macrophages cluster (MS4A7)(Zhou et al	-
Unique	2020a), and the result was visualized using Uniform Manifold Approximation and Projection (UMAP)	-
Unique	MS4A7 macrophages were colored according to its expression level of RhoA, allowing us to spatially	-
Unique	Western blotting After washing with PBS, tissues and cells were collected in RIPA lysis buffer	-
Unique	Proteins were separated by SDS-PAGE and transferred to polyvinylidene fluoride membranes (Cat#IPVH0010, Millipore, USA) according	-
Unique	Membranes were blocked with 5% skim milk for 1 h at RT, then cut horizontally	-
Unique	Membranes were incubated with primary antibody at 4°C overnight	-
Unique	To detect phosphorylated proteins at the same site, membranes were stripped using Stripping buffer (Cat#ab282569,	-
Unique	On the following day, horseradish peroxidase (HRP)-conjugated secondary antibodies were applied, and enhanced chemiluminescence was	-
Unique	Information on the antibodies utilized is provided in Supplementary materials Table 1	-
Unique	Statistical analyses Statistical analysis was performed using SPSS 25.0 software	-

Unique	Comparisons were made using the t-test, one-way ANOVA, or two-way ANOVA, followed by Tukey's post	-
Unique	Data are presented as mean ± S.D	-
Unique	(n ≥ 3), with P 0.05 considered statistically significant	-
Unique	Results 1	-
Unique	RhoA is upregulated in synovial macrophages and correlates with OA severity	-
Unique	To investigate the role of macrophage RhoA in OA progression and its correlation with disease	-
Unique	Samples were stratified based on OA severity determined by MRI grading (Fig	-
Unique	1A)	-
Unique	Bioinformatic analysis based on single-cell RNA sequencing data from the GEO database (GSE133449) indicated that	-
Unique	1B)	-
Unique	Immunofluorescence (IF) and Western blot (WB) analyses in the human samples confirmed that RhoA positive	-
Unique	1C, D)	-
Unique	In a mouse OA model, immunohistochemistry (IHC) revealed the RhoA positive intensity in the synovial	-
Unique	1E)	-
Unique	Furthermore, double immunofluorescent-staining identified that the RhoA expression in synovial is mainly localized in F4/80$^+$	-
Unique	1F)	-
Unique	Figure 1: RhoA expression in OA patients and mouse models	-
Unique	Representative MRI images of OA patients	-
Unique	The expression level of RhoA in macrophages was visualized using UMAP	-
Unique	C, D	-
Unique	IF and WB analysis of RhoA of positive cells rate and protein expression in ex	-
Unique	IHC for RhoA in synovium: percentage of RhoA$^+$ cells relative to total haematoxylin-stained nuclei	-
Unique	IF co-staining: percentage of RhoA$^+$ F4/80$^+$ double-positive cells relative to total DAPI$^+$ nuclei	-
Unique	*P 0.05	-
Unique	Generation of macrophage-specific RhoA conditional knockout mice	-
Unique	Previous studies on RhoA in OA relied heavily on pharmacological interventions, where observed effects could	-
Unique	To specifically investigate the role of macrophage RhoA upregulation in OA, we generated macrophage-specific RhoA	-

Unique	2A, B)	-
Unique	At 8 weeks of age, both cKO and control mice underwent OA modeling	-
Unique	WB and IF confirmed that RhoA was nearly undetectable in macrophages from cKO mice compared	-
Unique	2C, D)	-
Unique	These results validate the successful generation of macrophage-specific RhoA cKO mice for subsequent studies	-
Unique	Figure 2: Generation and validation of macrophage-specific RhoA conditional knockout mice	-
Unique	Schematic of the breeding strategy	-
Unique	Genotyping for identification of Lyz2Cre; RhoAflox/flox mice	-
Unique	WB analysis of RhoA knockout efficiency in primary macrophages	-
Unique	IF evaluation of RhoA expression in synovial macrophages 8 weeks post-DMM surgery	-
Unique	*P 0.05	-
Unique	Macrophage-specific RhoA knockout exacerbates cartilage matrix loss in OA	-
Unique	At 8 weeks post-OA modeling, Toluidine Blue and Safranin O staining revealed significantly greater cartilage	-
Unique	3A, B)	-
Unique	Hematoxylin and eosin (H&E) staining indicated increased synovial inflammation in the cKO group (Fig	-
Unique	3C)	-
Unique	Micro-CT analysis showed a marked increase in osteophyte volume, decreased trabecular number (Tb.N) and thickness	-
Unique	3D)	-
Unique	These findings suggest that deletion of macrophage RhoA promotes cartilage damage and subchondral bone loss,	-
Unique	Figure 3: Macrophage RhoA deletion exacerbates OA cartilage matrix destruction	-
Unique	A, B	-
Unique	Cartilage matrix loss assessed by Toluidine Blue and Safranin O staining	-
Unique	Synovial inflammation and cartilage integrity evaluated by H&E staining	-
Unique	Three-dimensional reconstruction and quantitative analysis of osteophyte volume and subchondral bone parameters (Tb.N, Tb.Th, Tb.Sp)	-
Unique	*P 0.05	-
Unique	RhoA ablation promotes M1 and suppresses M2 macrophage polarization	-
Unique	To determine whether RhoA regulates macrophage polarization, we performed WB and IF analyses	-
Unique	Macrophage-specific RhoA deletion significantly increased protein levels of the M1 markers iNOS and CD86, while	-

Unique	4A)	-
Unique	IF quantification confirmed a significant increase in iNOS signal intensity and a decrease in Arg-1	-
Unique	4B)	-
Unique	Together, these data indicate that RhoA ablation shifts macrophage polarization toward a pro-inflammatory M1 phenotype	-
Unique	Figure 4	-
Unique	Macrophage RhoA deletion promotes pro-inflammatory polarization	-
Unique	Representative WB and densitometric analysis of iNOS, Arg-1, and CD86 in macrophages from Cre and	-
Unique	Representative IF images and integrated fluorescence intensity of iNOS and Arg-1 in macrophages	-
Unique	*P 0.05	-
Unique	Conditioned medium from cKO macrophages accelerates senescence in mouse chondrocytes and human OA chondrocytes	-
Unique	To assess the impact of macrophage RhoA knockout on chondrocytes, we established a co-culture system	-
Unique	5A)	-
Unique	We conducted transcriptomic profiling using RNA sequencing (RNA-seq) with co-cultured mouse chondrocytes, and Gene Set	-
Unique	5B)	-
Unique	Consistent with the GSEA findings, WB analysis showed decreased LC3-II/I ratio, indicating the autophagy were	-
Unique	5C)	-
Unique	ROS and JC-1 staining demonstrated significantly higher reactive oxygen species (ROS) levels (Fig	-
Unique	5D) and a marked reduction in healthy mitochondria (JC-1 aggregates) in chondrocytes from the cKO	-
Unique	5E)	-
Unique	WB analysis revealed upregulation of iNOS, MMP13, p53, and p21 in chondrocytes from cKO group	-
Unique	5F-H)	-
Unique	Senescence-associated β-galactosidase (SA-β-gal) staining showed a significant increase in senescent chondrocytes in the cKO group	-
Unique	5I)	-
Unique	Similarly, the human OA chondrocytes co-cultured with cKO macrophages showed reduced mitochondrial membrane potential (lower	-
Unique	5J-M)	-
Unique	These data demonstrate that macrophage RhoA deletion enhances chondrocyte autophagy suppression, ROS production, and mitochondrial	-
Unique	Figure 5	-

Unique	Macrophage RhoA deletion exacerbates chondrocyte senescence	-
Unique	Schematic of the co-culture model	-
Unique	GSEA showing negative enrichment of the autophagy gene set in chondrocytes from the cKO group	-
Unique	WB analysis of the LC3B-II/I ratio in mouse chondrocytes	-
Unique	IF staining for ROS using DCFH-DA	-
Unique	JC-1 staining for mitochondrial membrane potential in mouse chondrocytes; aggregates (red) indicate healthy potential, monomers	-
Unique	F-H	-
Unique	WB analysis of iNOS, MMP13, p53, and p21 in mouse chondrocytes	-
Unique	Quantification of SA-β-gal-positive senescent mouse chondrocytes	-
Unique	JC-1 staining and quantitative analysis of mitochondrial membrane potential in human OA chondrocytes	-
Unique	K, L	-
Unique	WB analysis and SA-β-gal staining for senescence markers (p53, p21) and senescent cells in human	-
Unique	Safranin O and Toluidine Blue staining assessing proteoglycan loss in human OA cartilage explants	-
Unique	*P 0.05	-
Unique	IL-17C mediates the pro-senescent effect of macrophage RhoA deletion on chondrocytes	-
Unique	To identify mechanisms by which macrophage RhoA influences chondrocytes, we performed RNA-seq on cartilage from	-
Unique	Lyz-RhoA-cKO) and on mouse chondrocytes co-cultured with macrophages	-
Unique	Intersection analysis identified 193 common differentially expressed genes (Fig	-
Unique	6A)	-
Unique	KEGG enrichment analysis revealed significant enrichment of the IL-17 signaling pathway relevant to OA (Fig	-
Unique	6B), and GO analysis showed that the 'reactive oxygen species metabolic process' was among the	-
Unique	6C)	-
Unique	ELISA of joint fluid from OA mice and macrophages supernatants, along with WB analysis of	-
Unique	6D, E	-
Unique	Fig	-
Unique	1A, B)	-
Unique	Furthermore, COX2, a key effector of IL-17 signaling, was elevated in chondrocytes co-cultured with cKO	-
Unique	6F)	-

Unique	These results suggest that macrophage RhoA deletion activates the IL-17 signaling pathway in chondrocytes via	-
Unique	To further validate IL-17C's role, the IL-17C-neutralizing antibody was added into the co-culture system in	-
Unique	Fig 1C), and was administered via intra-articular injection to cKO mice (10mg/kg)	-
Unique	WB showed reduced expression of iNOS, MMP13, p53, and p21 in chondrocytes from the cKO	-
Unique	6G)	-
Unique	Micro-CT analysis revealed that Anti-IL-17C injection significantly reduced osteophyte formation in cKO mice (Fig	-
Found	6H)	sandvik.coromant.com sandvik.coromant.com amazon.com
Unique	Safranin O and Toluidine Blue staining demonstrated that Anti-IL-17C mitigated cartilage matrix destruction induced by	-
Unique	6I, J)	-
Unique	These results indicate that macrophage RhoA deletion accelerates cellular senescence and OA progression via enhanced	-
Unique	Given that macrophage RhoA deletion drives OA through excessive IL-17C secretion, we explored combining an	-
Unique	CT04 (a RhoA activity inhibitor) alone or CT04 Anti-IL-17C was added into the co-culture system	-
Unique	Micro-CT showed a significant reduction in osteophyte volume in the CT04 Anti-IL-17C group compared to	-
Unique	6K)	-
Unique	Toluidine Blue staining revealed less cartilage matrix damage in mice treated with the combination therapy	-
Unique	6L)	-
Unique	Similarly, Safranin O and Toluidine Blue staining showed the matrix loss in explants was attenuated	-
Unique	6M)	-
Unique	These results indicate that IL-17C neutralization effectively blocks the detrimental effects of macrophage RhoA deletion	-
Found	Figure 6	amazon.com shutterstock.com
Unique	IL-17C mediates macrophage RhoA-induced chondrocyte senescence	-
Unique	A-C	-
Unique	Integrated transcriptome analysis of mouse OA cartilage (Red) and co-cultured chondrocytes (Yellow)	-
Unique	IL-17C secretion in joint fluid and macrophage supernatants measured by ELISA	-
Unique	WB analysis of IL-17C in macrophages	-
Unique	WB analysis of COX2 in co-cultured chondrocytes	-

Unique	WB analysis of iNOS, MMP13, p53, and p21 in co-cultured chondrocytes after Anti-IL-17C treatment	-
Unique	Micro-CT 3D reconstruction and quantification of osteophyte volume in OA mice	-
Unique	I, J	-
Unique	Safranin O and Toluidine Blue staining assessing cartilage matrix loss in human OA explants	-
Unique	Micro-CT analysis of osteophyte volume in OA mice	-
Unique	Toluidine Blue staining of cartilage matrix in OA mice	-
Unique	Safranin O and Toluidine Blue staining of matrix loss in human OA explants	-
Unique	*P 0.05	-
Unique	IL-17C promotes chondrocyte senescence by activating the PI3K/AKT/mTOR axis	-
Unique	RNA-seq of chondrocytes revealed significant enrichment of the PI3K-AKT-mTOR signaling pathway among upregulated WikiPathways (Fig	-
Unique	7A)	-
Unique	Given prior reports indicate that (i) IL-17 can activate PI3K-AKT-mTOR signaling(Faust et al	-
Unique	2020; Zhou et al	-
Unique	2020b), (ii) mTOR activation suppresses autophagy(Li et al	-
Found	2025; Qiu et al	frontiersin.org
Unique	2025), and (iii) autophagy inhibition promotes senescence(Chen et al	-
Found	2025; Feng et al	arxiv.org assets.amazon.science
Unique	2024), we hypothesized that IL-17C links these events to accelerate cartilage senescence	-
Unique	WB analysis confirmed significantly increased levels of PI3K, phosphorylated AKT (p-AKT), and phosphorylated mTOR (p-mTOR)	-
Unique	7B)	-
Unique	Chondrocytes co-cultured with cKO macrophages showed suppressed autophagy (lower LC3B-II/I ratio), elevated inflammation (iNOS/MMP13) and	-
Unique	7 C-F)	-
Unique	In co-cultured human OA chondrocytes, PI3K inhibition similarly reduced iNOS expression, ROS levels, p53/p21 protein	-
Unique	7 G-J)	-
Unique	Collectively, these results indicate that macrophage RhoA deletion promotes chondrocyte senescence via the IL-17C-PI3K-AKT-mTOR signaling	-
Unique	Figure 7	-
Unique	IL-17C accelerates chondrocyte senescence via PI3K/AKT/mTOR activation	-

Unique	WikiPathways enrichment analysis of RNA-seq data from chondrocytes co-cultured with macrophages	-
Unique	WB analysis of PI3K, AKT, mTOR, p-AKT, and p-mTOR in co-cultured chondrocytes	-
Unique	C-F	-
Unique	WB and SA-β-gal assays quantifying LC3B, iNOS, MMP13, p53, p21, and senescent mouse chondrocytes	-
Unique	G, H	-
Unique	IF analysis of iNOS and ROS in human OA chondrocytes	-
Unique	I, J	-
Unique	WB and SA-β-gal assays quantifying p53, p21, and senescent human OA chondrocytes	-
Unique	*P 0.05	-
Unique	RhoA regulates IL-17C expression in macrophages via the LATS/YAP/CCN2 axis	-
Unique	To elucidate how macrophage RhoA regulates OA progression, we first examined its canonical downstream effector	-
Unique	WB analysis showed that macrophage-specific RhoA deletion did not significantly alter ROCK expression in macrophages	-
Unique	8A), suggesting that RhoA acts independently of ROCK in this context	-
Unique	RNA-seq of primary macrophages from control and cKO mice revealed that RhoA deletion upregulated 1775	-
Unique	8 B-D)	-
Unique	KEGG analysis of differentially expressed genes identified the top 20 enriched pathways, with literature review	-
Unique	8E)	-
Unique	WB analysis showed a significant upregulation of phosphorylated LATS (p-LATS), phosphorylated YAP (p-YAP), together with	-
Unique	Treatment with the Hippo pathway inhibitor XMU-MP-1 (400μM)(Li et al	-
Unique	2024) significantly reduced IL-17C protein expression and secretion in cKO macrophages, as shown by WB	-
Unique	8 F-J)	-
Unique	Co-culture of human OA chondrocyte explants with XMU-MP-1-treated cKO macrophages resulted in reduced matrix loss	-
Unique	8K)	-
Unique	These results suggest that RhoA deletion in macrophages promotes IL-17C secretion and exacerbates OA progression	-
Unique	Figure 8: Macrophage RhoA regulates IL-17C secretion via the LATS/YAP/CCN2 signaling axis	-
Unique	WB analysis of ROCK protein levels in macrophages	-
Unique	B-F	-

Unique	RNA-seq analysis of Cre vs	-
Unique	cKO macrophages	-
Found	Volcano plot of differentially expressed genes (DEGs)	researchgate.net
Unique	Heatmap of DEGs across samples	-
Unique	D, E	-
Unique	KEGG enrichment analysis of DEGs	-
Unique	F-I	-
Unique	WB analysis of LATS, YAP, their phosphorylated forms, CCN2 and IL-17C in macrophages	-
Unique	ELISA of IL-17C in macrophage supernatants	-
Unique	Safranin O and Toluidine Blue staining assessing matrix loss in human OA cartilage explants	-
Unique	*P 0.05	-
Found	N.S	abbreviations.com en.wikipedia.org lsd.law
Unique	: not significant	-
Unique	Discussion This study reveals a novel and critical role for macrophage-specific RhoA signaling in alleviating	-
Unique	2024; Jiang et al	-
Unique	2023)	-
Unique	While previous literature has firmly established RhoA/ROCK as a promoter of cartilage catabolism and subchondral	-
Found	2023; Zhu et al	link.springer.com
Unique	2013), our findings paradoxically demonstrate that RhoA cKO in the synovial macrophage compartment exacerbates OA	-
Unique	This cell-type-specific function underscores the complex, context-dependent nature of RhoA signaling in the joint and	-
Unique	As key players in the innate immune response within the joint cavity, synovial macrophages respond	-
Found	2022; Yin et al	agupubs.onlinelibrary.wiley.com
Unique	2024; Zhao et al	-
Unique	2023)	-
Unique	This suggests three potential strategies for targeting the detrimental effects of synovial macrophages: (1) blocking	-
Found	2007; Hamasaki et al	researchgate.net
Found	2020; Zhang et al	researchgate.net
Unique	2018); (2) targeting the action pathways by identifying and neutralising secreted pathogenic factors(Liu et al	-
Unique	2023; Mikulkova et al	-

Unique	2024); (3) intervening at downstream targets by understanding changes in chondrocyte receptors and signalling pathways	-
Unique	2021)	-
Unique	Present key finding is the identification of IL-17C as the primary effector links the cross-talk	-
Unique	The IL-17 family, particularly IL-17A, has been implicated in OA(Faust et al	-
Unique	2020); however, the role of IL-17C is still unknow	-
Unique	The evidences of current study demonstrate that RhoA acts as a transcriptional brake on IL-17C	-
Unique	This macrophage-chondrocyte crosstalk via the RhoA/IL-17C axis represents a significant conceptual advance in understanding OA	-
Unique	Another interesting finding of this study is that RhoA deletion in macrophages activated YAP/CCN2 signaling	-
Unique	Previous studies have indicated that RhoA, upon oxidation by mitochondrial superoxide, activates YAP/TAZ, leading to	-
Unique	2024)	-
Unique	It has been reported that CCN2 enhances IL-17 synthesis by disrupting the inhibitory effect of	-
Unique	2024)	-
Unique	This shifts the paradigm away from the classic RhoA-ROCK cytoskeletal axis and towards the Hippo/YAP	-
Unique	Which suggests that therapeutic strategies targeting RhoA for OA treatment must be highly cell-type-specific, as	-
Found	For example, Allen et al	researchgate.net link.springer.com
Unique	demonstrated that opposing RhoA actions in spinal neurons versus astrocytes restrict regeneration; only neuron-specific RhoA	-
Unique	2021)	-
Unique	Second, this study indicates IL-17C as a promising and more tractable therapeutic target	-
Unique	The synergistic benefit observed when combining a RhoA activator (CT04) with an IL-17C-neutralizing antibody provides	-
Unique	In conclusion, our results demonstrate a previously unknown RhoA/YAP/IL-17C signaling axis in synovial macrophages that	-
Unique	By IL-17C paracrine, macrophage RhoA plays important role in maintaining chondrocyte homeostasis and cartilage integrity	-
Unique	This study not only redefines the function of RhoA in OA but also opens new	-

Top plagiarizing domains: **researchgate.net** (5 matches); **agupubs.onlinelibrary.wiley.com** (2 matches); **frontiersin.org** (2 matches); **sandvik.coromant.com** (2 matches); **amazon.com** (2 matches); **link.springer.com** (2 matches); **sciencedirect.com** (1 matches); **pmc.ncbi.nlm.nih.gov** (1 matches); **jstor.org** (1 matches); **ajol.info** (1 matches); **shutterstock.com** (1 matches); **arxiv.org** (1 matches); **assets.amazon.science** (1 matches); **abbreviations.com** (1 matches); **en.wikipedia.org** (1 matches); **lsd.law** (1 matches);

Abstract Objective: The GTPase RhoA is known as a regulator involved in cartilage degeneration and subchondral bone remodeling related to osteoarthritis (OA). However, its specific role in synovial macrophages, the key immune cells of OA related tissues, remains entirely unexplored. Methods: Herein, the RhoA expression in human and mouse OA synovium was analyzed. A macrophage-specific RhoA conditional knockout (cKO) mouse model was generated. Histological staining, OARSI scoring, and micro-CT were used to assess cartilage damage, while Western blot, immunofluorescence staining, and ELISA assessed changes in cellular function. Transcriptome sequencing and validation of signaling pathways were conducted using tissues and cells from patients with OA and OA mice. Results: The collected results indicate that RhoA expression was significantly upregulated in synovial macrophages from OA patients and mice, correlating with disease severity. Contrary to its reported role in chondrocytes or endothelial cells, macrophage-specific RhoA deletion exacerbated OA, demonstrating enhanced cartilage destruction, subchondral bone loss, and synovitis. RhoA-deficient macrophages exhibited a pro-inflammatory M1 polarization and secreted high levels of IL-17C. This cytokine was necessary and sufficient to induce chondrocyte senescence, as evidenced by increased p53/p21, ROS, mitochondrial dysfunction, and suppressed autophagy, via activation of the PI3K/AKT/mTOR pathway. Mechanistically, RhoA ablation in macrophages activated the Hippo pathway effectors YAP/CCN2, leading to IL-17C transcription, independently of the canonical ROCK pathway. Conclusion: In conclusion, present study reveals a previously unrecognized, protective role for macrophage RhoA in OA. It functions as a critical brake on a novel YAP-IL-17C axis, thereby preserving chondrocyte. This study redefines RhoA's role in joint homeostasis and nominates IL-17C as a potential therapeutic target for OA. Keywords: Osteoarthritis; RhoA; Synovial Macrophage; Chondrocyte Senescence; YAP/IL-17C Introduction Osteoarthritis (OA) is a highly prevalent and debilitating degenerative joint disease, affecting 600 million people worldwide(Duong et al. 2023; Hunter and Bierma-Zeinstra 2019; Roelofs and De Bari 2024). OA is well known as its progressive synovial inflammation, cartilage deterioration, and subchondral bone remodeling, however, the molecular drivers of OA pathogenesis remain incompletely elucidated, hindering the development of efficient therapeutic strategies(Jia et al. 2024; Kloppenburg 2023; Xu et al. 2023). Among the various signaling pathways implicated in OA, the Rho GTPase RhoA has garnered increasing attention(Deng et al. 2019; Novakofski et al. 2009). Accumulating evidence positions RhoA as a central regulator of cytoskeletal dynamics, cell differentiation, and inflammatory responses within the joint. Studies have documented its pathogenic role in promoting chondrocyte hypertrophy, cartilage matrix degradation, and vascular dysfunction in subchondral bone, highlighting RhoA as a potential therapeutic target(Sui et al. 2022; Wang and Beier 2005; Wang et al. 2004; Zhang et al. 2025). However, the existing researches primarily focused on RhoA functions in chondrocytes and endothelial cells(He et al. 2024; Jiang et al. 2023). The role of RhoA in synovial macrophages, key sentinels and amplifiers of joint inflammation, has never been reported. Given that macrophage polarization critically influences OA progression(Cai et al. 2025; Jiang et al. 2025; Wang et al. 2025), we hypothesized that RhoA signaling within these immune cells could be a pivotal regulator of the synovial environment and cartilage homeostasis. In this study, we systematically investigated the

expression and functional significance of RhoA in synovial macrophages during OA. Combined with clinical OA samples, experimental OA model in wild type mice and macrophage-specific conditional knockout (cKO) mice, and in vitro culture systems, the present findings uncovered an unexpected role of that RhoA upregulation in OA synovial macrophages exerts a protective, cell-type-specific function in OA. Mechanically, macrophages RhoA modulates IL-17C secretion through the non-canonical Hippo/YAP signaling pathway, and the IL-17C paracrine drives chondrocyte senescence via the PI3K/AKT/mTOR pathway. Present study not only broaden the understanding of RhoA in OA but also reveal a previously unrecognized crosstalk mechanism between synovial macrophages and articular chondrocytes. Materials and methods Experimental animals and human OA samples Animals：Male C57BL/6 mice (8 weeks, 20–22 g, SPF) were purchased from the Southern Medical University Animal Center (Guangzhou, China) and maintained at 22 ± 2 °C, 55 ± 10 % humidity and a 12-h light/dark cycle. OA was induced by destabilization of the medial meniscus (DMM) at 8 weeks of age. For primary chondrocyte isolation, 3-day-old neonatal pups were euthanized by decapitation. All procedures were approved by the Institutional Animal Care and Use Committee of Southern Medical University (approval no. SMU-L2018158). Transgenic mice：Lyz2-Cre mice (Sangon Biotech, Suzhou, China) were backcrossed to C57BL/6 for ⩾ 6 generations and then crossed with RhoAflox/flox mice to produce macrophage-specific (Lyz2-Cre; RhoAflox/flox) RhoA knockouts. Cre-negative RhoAflox/flox littermates served as controls. Genotyping was performed following the protocols described in our previous publication(Xu et al. 2021). Human tissues：Synovial samples were obtained from patients with end-stage OA (KL grade 1–4; mean age 67 ± 5) undergoing total knee replacement and from trauma-amputation controls (mean age 35 ± 10) at the Ganzhou Hospital–Nanfang Hospital. The study protocol was approved by the Institutional Review Board (approval no. TY-ZKY2024-141-01), and written informed consent was obtained from all participants. Primary cell and OA cartilage culture Primary chondrocytes were isolated from 3-day-old C57BL/6 pups by enzymatic digestion (0.2 % type II Collagenase, 2 h, 37 °C) and cultured in DMEM/F-12 with 10 % FBS and 1 % penicillin–streptomycin; cells were used at passage ⩽ 2. Bone marrow–derived macrophages (BMDMs) were generated by flushing femora and tibiae from 8-week-old mice and culturing in DMEM containing 10 % FBS and 20 ng/mL recombinant murine M-CSF (PeproTech, 315-02) for 7 days; purity was 95 %(Zou et al. 2021). Human OA cartilage was obtained from total-knee-replacement patients within 2 h of surgery, minced into 1 cm^2 explants and cultured in DMEM/F-12 plus 10 % FBS at 37 °C, 5 % CO_2(Xu et al. 2023). All human tissues were collected under approval TY-ZKY2024-141-01 with written informed consent. Animal surgery and drug administration OA was induced by destabilisation of the medial meniscus (DMM) in the right knee of 8-week-old male C57BL/6 mice. Under sterile conditions and continuous isoflurane anaesthesia (2 % in O_2, 1 L/min), the medial meniscotibial ligament was transected under a dissecting microscope (× 10). Sham mice underwent identical arthrotomy without ligament transection(Xu et al. 2023). Post-operative analgesia (Carprofen 5 mg/kg s.c. every 12 h for 48 h) and prophylactic antibiotics (enrofloxacin 5 mg/kg s.c. once daily for 3 days) were administered. Beginning 7 days after surgery, mice received weekly intra-articular injections (30-gauge needle via the patellar tendon, 9–11 a.m.) for 7 consecutive weeks: (1) DMM CT04: CT04 (1 μg/μL, 10 μL(Zhang et al. 2012)) in 0.9 % NaCl, pH

7.4. (2) DMM CT04 Anti-IL-17C: CT04 (2 μg/μL, 5 μL) plus Anti-IL-17C (20 mg/kg, 5 μL in 0.9 % NaCl(Zhou et al. 2020b)). (3) DMM vehicle (Control group): equal volume of sterile 0.9 % NaCl. Stock solutions were sterile-filtered (0.22 μm), aliquoted, and stored at −80 °C. Aliquots were thawed at 4 °C immediately before use, vortexed for 5 s, centrifuged 1 min at 4 °C, and kept on ice ⩽ 2 h prior to injection. Micro-computed tomography The mouse knee tissues were preserved in a 4% paraformaldehyde solution for 72 hours before undergoing micro-computed tomography (micro-CT) analysis, following established methodologies. The analysis assessed several parameters, including the osteophyte count, trabecular separation (Tb. Sp, mm), trabecular thickness (Tb.Th, mm), and trabecular number (Tb. N, 1/mm). These parameters were quantified using the accompanying software for the micro-CT system (ZKKS-MicroCT4.1, China). Histological staining Knee joints were fixed in 4 % paraformaldehyde for 24 h at 4 °C, decalcified in 10 % (w/v) EDTA (pH 7.4, 4 °C, 50 days), dehydrated through ascending ethanol (70 %, 80 %, 90 %, 100 %, each 30 min), embedded in paraffin and sectioned into 5 μm. Safranin O-fast green (0.1 % Safranin O, 5 min; 0.2 % Fast Green, 5 min) and Toluidine blue (0.04 %, 2 min) staining were performed. Cartilage degeneration was scored according to OARSI 2019 guidelines (0–6 scale) by two blinded observers. Synovitis was graded on H&E-stained sections (0–9 scale). For immunohistochemistry, sections were deparaffinized, rehydrated, subjected to heat-mediated antigen retrieval (citrate buffer, pH 6.0, 95 °C, 20 min), incubated with 3 % H_2O_2 (15 min) and blocked with goat serum (30 min). Primary antibodies (dilutions in Table S1) were incubated overnight at 4 °C, followed by HRP-conjugated secondary antibody at room temperature (RT, 1h) and DAB (⩽ 3 min). Sections were counterstained with haematoxylin, dehydrated and mounted. For immunofluorescence, antigen retrieval and blocking (5 % normal goat serum, 1 h) were performed as above. Primary antibodies were incubated overnight at 4 °C, followed by Alexa Fluor-conjugated secondary antibodies (488 or 568, 2 h, RT). Nuclei were stained with DAPI (1 μg/mL, 5 min). Images were captured using a Nikon A1 confocal microscope at identical settings. The list of antibodies used is provided in the Supplementary Materials Table 1. RNA sequencing Total RNA was extracted using Trizol Reagent (Invitrogen Life Technologies), with concentration, quality, and integrity assessed via a NanoDrop spectrophotometer (Thermo Scientific). Three micrograms of RNA were used for library preparation. mRNA was isolated from total RNA using poly-T oligo-attached magnetic beads, followed by fragmentation using divalent cations in an Illumina proprietary buffer at elevated temperature. First-strand cDNA was synthesized with random primers and Super-Script II, and second-strand synthesis was performed using DNA Polymerase I and RNase H. Exonuclease/polymerase activities converted remaining overhangs into blunt ends, and the enzymes were removed. Following adenylation of the 3′ ends, Illumina PE adapter oligonucleotides were ligated for hybridization preparation. To select cDNA fragments of 400-500 bp, the library was purified using the AMPure XP system (Beckman Coulter, Beverly, CA, USA). Adapter-ligated DNA fragments were enriched through 15 cycles of PCR using Illumina PCR Primer Cocktail. The products were purified (AMPure XP system) and quantified using the Agilent high-sensitivity DNA assay on a Bioanalyzer 2100 system (Agilent). The sequencing library was processed on the NovaSeq 6000 platform (Illumina) at Shanghai Personal Biotechnology Cp. Ltd. Transcriptome analysis followed the manufacturer's protocol. Single-cell RNA sequencing data processing The single-cell RNA

sequencing dataset GSE133449(Sun et al. 2020) (https://doi.org/ 10.1136/annrheumdis-2019-215926) was processed using the Seurat R package. These data were originated from the patients with OA (n = 4). Initial quality control steps included filtering cells with high mitochondrial gene content (20%) and low gene count (200 genes per cell). Normalization and scaling of the data were performed, followed by the identification of highly variable genes across cells. A differential expression analysis was performed on macrophages cluster (MS4A7)(Zhou et al. 2020a), and the result was visualized using Uniform Manifold Approximation and Projection (UMAP). MS4A7 macrophages were colored according to its expression level of RhoA, allowing us to spatially observe the distribution of gene expression within the UMAP embedding. Western blotting After washing with PBS, tissues and cells were collected in RIPA lysis buffer (Cat#FD009, Fdbio Science, China) containing phosphatase and protease inhibitors, followed by protein extraction through lysis at RT for 30 min. Proteins were separated by SDS-PAGE and transferred to polyvinylidene fluoride membranes (Cat#IPVH0010, Millipore, USA) according to standard protocols. Membranes were blocked with 5% skim milk for 1 h at RT, then cut horizontally based on molecular weight, as labelled by Precision Plus Protein (10 - 250 kD, Cat#1610374S, Bio-Rad, USA). Membranes were incubated with primary antibody at 4°C overnight. To detect phosphorylated proteins at the same site, membranes were stripped using Stripping buffer (Cat#ab282569, Abcam, UK), re-blocked with 5% skim milk for 1 h at RT, and incubated with primary antibody overnight at 4 °C. On the following day, horseradish peroxidase (HRP)-conjugated secondary antibodies were applied, and enhanced chemiluminescence was used to detect target protein expression levels. Information on the antibodies utilized is provided in Supplementary materials Table 1. Statistical analyses Statistical analysis was performed using SPSS 25.0 software. Comparisons were made using the t-test, one-way ANOVA, or two-way ANOVA, followed by Tukey's post hoc test. Data are presented as mean ± S.D. (n ⩾ 3), with P 0.05 considered statistically significant. Results 1. RhoA is upregulated in synovial macrophages and correlates with OA severity. To investigate the role of macrophage RhoA in OA progression and its correlation with disease severity, synovial tissues were collected from OA patients during surgery. Samples were stratified based on OA severity determined by MRI grading (Fig. 1A). Bioinformatic analysis based on single-cell RNA sequencing data from the GEO database (GSE133449) indicated that RhoA was highly expressed in macrophages within OA tissues (Fig. 1B). Immunofluorescence (IF) and Western blot (WB) analyses in the human samples confirmed that RhoA positive cells rate and protein levels in synovial tissues increased with OA severity (Fig. 1C, D). In a mouse OA model, immunohistochemistry (IHC) revealed the RhoA positive intensity in the synovial is gradually increased at 4 and 8 weeks post-surgery (Fig. 1E). Furthermore, double immunofluorescent-staining identified that the RhoA expression in synovial is mainly localized in F4/80$^+$ macrophages (Fig. 1F). Figure 1: RhoA expression in OA patients and mouse models. A. Representative MRI images of OA patients. B. The expression level of RhoA in macrophages was visualized using UMAP. C, D. IF and WB analysis of RhoA of positive cells rate and protein expression in ex vivo human OA synovial tissues. E. IHC for RhoA in synovium: percentage of RhoA$^+$ cells relative to total haematoxylin-stained nuclei. F. IF co-staining: percentage of RhoA$^+$ F4/80$^+$ double-positive cells relative to total DAPI$^+$ nuclei. *P 0.05. 2. Generation of macrophage-specific RhoA conditional knockout mice. Previous studies

on RhoA in OA relied heavily on pharmacological interventions, where observed effects could stem from both direct and indirect actions across multiple cell types. To specifically investigate the role of macrophage RhoA upregulation in OA, we generated macrophage-specific RhoA conditional knockout mice (Lyz2Cre; RhoAflox/flox, cKO) by crossing Lyz2Cre mice with RhoAflox/flox mice; littermate Lyz2Cre mice served as controls (Fig. 2A, B). At 8 weeks of age, both cKO and control mice underwent OA modeling. WB and IF confirmed that RhoA was nearly undetectable in macrophages from cKO mice compared to controls (Fig. 2C, D). These results validate the successful generation of macrophage-specific RhoA cKO mice for subsequent studies. Figure 2: Generation and validation of macrophage-specific RhoA conditional knockout mice. A. Schematic of the breeding strategy. B. Genotyping for identification of Lyz2Cre; RhoAflox/flox mice. C. WB analysis of RhoA knockout efficiency in primary macrophages. D. IF evaluation of RhoA expression in synovial macrophages 8 weeks post-DMM surgery. *P 0.05. 3. Macrophage-specific RhoA knockout exacerbates cartilage matrix loss in OA. At 8 weeks post-OA modeling, Toluidine Blue and Safranin O staining revealed significantly greater cartilage matrix destruction in cKO mice compared to controls (Fig. 3A, B). Hematoxylin and eosin (H&E) staining indicated increased synovial inflammation in the cKO group (Fig. 3C). Micro-CT analysis showed a marked increase in osteophyte volume, decreased trabecular number (Tb.N) and thickness (Tb.Th), and increased trabecular separation (Tb.Sp) in cKO mice (Fig. 3D). These findings suggest that deletion of macrophage RhoA promotes cartilage damage and subchondral bone loss, thereby exacerbating OA progression. Figure 3: Macrophage RhoA deletion exacerbates OA cartilage matrix destruction. A, B. Cartilage matrix loss assessed by Toluidine Blue and Safranin O staining. C. Synovial inflammation and cartilage integrity evaluated by H&E staining. D. Three-dimensional reconstruction and quantitative analysis of osteophyte volume and subchondral bone parameters (Tb.N, Tb.Th, Tb.Sp) by micro-CT. *P 0.05. 4. RhoA ablation promotes M1 and suppresses M2 macrophage polarization. To determine whether RhoA regulates macrophage polarization, we performed WB and IF analyses. Macrophage-specific RhoA deletion significantly increased protein levels of the M1 markers iNOS and CD86, while reducing the M2 marker Arg-1, compared to controls (Fig. 4A). IF quantification confirmed a significant increase in iNOS signal intensity and a decrease in Arg-1 in cKO macrophages (Fig. 4B). Together, these data indicate that RhoA ablation shifts macrophage polarization toward a pro-inflammatory M1 phenotype and away from an anti-inflammatory M2 phenotype. Figure 4. Macrophage RhoA deletion promotes pro-inflammatory polarization. A. Representative WB and densitometric analysis of iNOS, Arg-1, and CD86 in macrophages from Cre and cKO mice. B. Representative IF images and integrated fluorescence intensity of iNOS and Arg-1 in macrophages. *P 0.05. 5. Conditioned medium from cKO macrophages accelerates senescence in mouse chondrocytes and human OA chondrocytes. To assess the impact of macrophage RhoA knockout on chondrocytes, we established a co-culture system in which Cre or cKO macrophages were incubated with either wild-type (WT) primary mouse chondrocytes or human OA cartilage explants/chondrocytes, according to the schematic design (Fig. 5A). We conducted transcriptomic profiling using RNA sequencing (RNA-seq) with co-cultured mouse chondrocytes, and Gene Set Enrichment Analysis (GSEA) revealed negative enrichment (NES = -1.99, FDR = 0.003) of an autophagy gene set in cKO group (Fig. 5B).

Consistent with the GSEA findings, WB analysis showed decreased LC3-II/I ratio, indicating the autophagy were reduced in chondrocytes co-cultured with cKO macrophages compared with the control group (Fig. 5C). ROS and JC-1 staining demonstrated significantly higher reactive oxygen species (ROS) levels (Fig. 5D) and a marked reduction in healthy mitochondria (JC-1 aggregates) in chondrocytes from the cKO group (Fig. 5E). WB analysis revealed upregulation of iNOS, MMP13, p53, and p21 in chondrocytes from cKO group (Fig. 5F-H). Senescence-associated β-galactosidase (SA-β-gal) staining showed a significant increase in senescent chondrocytes in the cKO group (Fig. 5I). Similarly, the human OA chondrocytes co-cultured with cKO macrophages showed reduced mitochondrial membrane potential (lower JC-1 red/green ratio), elevated senescence markers (p53, p21, SA-β-gal), and accelerated cartilage matrix loss compared with control group, as shown by Safranin O and Toluidine Blue staining (Fig. 5J-M). These data demonstrate that macrophage RhoA deletion enhances chondrocyte autophagy suppression, ROS production, and mitochondrial injury, thereby accelerating chondrocyte senescence. Figure 5. Macrophage RhoA deletion exacerbates chondrocyte senescence. A. Schematic of the co-culture model. B. GSEA showing negative enrichment of the autophagy gene set in chondrocytes from the cKO group. C. WB analysis of the LC3B-II/I ratio in mouse chondrocytes. D. IF staining for ROS using DCFH-DA. E. JC-1 staining for mitochondrial membrane potential in mouse chondrocytes; aggregates (red) indicate healthy potential, monomers (green) indicate depolarization. F-H. WB analysis of iNOS, MMP13, p53, and p21 in mouse chondrocytes. I. Quantification of SA-β-gal-positive senescent mouse chondrocytes. J. JC-1 staining and quantitative analysis of mitochondrial membrane potential in human OA chondrocytes. K, L. WB analysis and SA-β-gal staining for senescence markers (p53, p21) and senescent cells in human OA chondrocytes, respectively. M. Safranin O and Toluidine Blue staining assessing proteoglycan loss in human OA cartilage explants. *P 0.05. 6. IL-17C mediates the pro-senescent effect of macrophage RhoA deletion on chondrocytes. To identify mechanisms by which macrophage RhoA influences chondrocytes, we performed RNA-seq on cartilage from OA mice (Lyz-Cre vs. Lyz-RhoA-cKO) and on mouse chondrocytes co-cultured with macrophages. Intersection analysis identified 193 common differentially expressed genes (Fig. 6A). KEGG enrichment analysis revealed significant enrichment of the IL-17 signaling pathway relevant to OA (Fig. 6B), and GO analysis showed that the 'reactive oxygen species metabolic process' was among the significantly enriched biological processes (Fig. 6C). ELISA of joint fluid from OA mice and macrophages supernatants, along with WB analysis of macrophages, showed that the secretion and expression of IL-17C, but not IL-17A or IL-17B, were significantly upregulated in the cKO group (Fig. 6D, E. S. Fig. 1A, B). Furthermore, COX2, a key effector of IL-17 signaling, was elevated in chondrocytes co-cultured with cKO macrophages compared to the control group (Fig. 6F). These results suggest that macrophage RhoA deletion activates the IL-17 signaling pathway in chondrocytes via IL-17C secretion, influencing ROS metabolism and thus promoting OA. To further validate IL-17C's role, the IL-17C-neutralizing antibody was added into the co-culture system in which cKO macrophages were incubated with WT primary mouse chondrocytes or human OA cartilage explants,(100μM, S. Fig 1C), and was administered via intra-articular injection to cKO mice (10mg/kg). WB showed reduced expression of iNOS, MMP13, p53, and p21 in chondrocytes from the cKO Anti-IL-17C group compared to the cKO alone (Fig. 6G). Micro-CT analysis

revealed that Anti-IL-17C injection significantly reduced osteophyte formation in cKO mice (Fig. 6H). Safranin O and Toluidine Blue staining demonstrated that Anti-IL-17C mitigated cartilage matrix destruction induced by cKO macrophages in human OA explants (Fig. 6I, J). These results indicate that macrophage RhoA deletion accelerates cellular senescence and OA progression via enhanced IL-17 signaling. Given that macrophage RhoA deletion drives OA through excessive IL-17C secretion, we explored combining an IL-17C-neutralizing antibody with RhoA inhibition. CT04 (a RhoA activity inhibitor) alone or CT04 Anti-IL-17C was added into the co-culture system in which Cre macrophages were incubated with human OA cartilage explants, and was administered via intra-articular injection to Cre mice. Micro-CT showed a significant reduction in osteophyte volume in the CT04 Anti-IL-17C group compared to CT04 alone (Fig. 6K). Toluidine Blue staining revealed less cartilage matrix damage in mice treated with the combination therapy (Fig. 6L). Similarly, Safranin O and Toluidine Blue staining showed the matrix loss in explants was attenuated in the combination group (Fig. 6M). These results indicate that IL-17C neutralization effectively blocks the detrimental effects of macrophage RhoA deletion and enhances the therapeutic efficacy of RhoA inhibition in OA. Figure 6. IL-17C mediates macrophage RhoA-induced chondrocyte senescence. A-C. Integrated transcriptome analysis of mouse OA cartilage (Red) and co-cultured chondrocytes (Yellow). D. IL-17C secretion in joint fluid and macrophage supernatants measured by ELISA. E. WB analysis of IL-17C in macrophages. F. WB analysis of COX2 in co-cultured chondrocytes. G. WB analysis of iNOS, MMP13, p53, and p21 in co-cultured chondrocytes after Anti-IL-17C treatment. H. Micro-CT 3D reconstruction and quantification of osteophyte volume in OA mice. I, J. Safranin O and Toluidine Blue staining assessing cartilage matrix loss in human OA explants. K. Micro-CT analysis of osteophyte volume in OA mice. L. Toluidine Blue staining of cartilage matrix in OA mice. M. Safranin O and Toluidine Blue staining of matrix loss in human OA explants. *P 0.05. 7. IL-17C promotes chondrocyte senescence by activating the PI3K/AKT/mTOR axis. RNA-seq of chondrocytes revealed significant enrichment of the PI3K-AKT-mTOR signaling pathway among upregulated WikiPathways (Fig. 7A). Given prior reports indicate that (i) IL-17 can activate PI3K-AKT-mTOR signaling(Faust et al. 2020; Zhou et al. 2020b), (ii) mTOR activation suppresses autophagy(Li et al. 2025; Qiu et al. 2025), and (iii) autophagy inhibition promotes senescence(Chen et al. 2025; Feng et al. 2024), we hypothesized that IL-17C links these events to accelerate cartilage senescence. WB analysis confirmed significantly increased levels of PI3K, phosphorylated AKT (p-AKT), and phosphorylated mTOR (p-mTOR) in chondrocytes co-cultured with cKO macrophages compared to the control group (Fig. 7B). Chondrocytes co-cultured with cKO macrophages showed suppressed autophagy (lower LC3B-II/I ratio), elevated inflammation (iNOS/MMP13) and senescence (increased p53/p21 and SA-β-gal-positive cells), which were markedly attenuated by the treatment with a PI3K inhibitor (Fig. 7 C-F). In co-cultured human OA chondrocytes, PI3K inhibition similarly reduced iNOS expression, ROS levels, p53/p21 protein abundance, and the proportion of SA-β-gal-positive cells compared to the cKO group (Fig. 7 G-J). Collectively, these results indicate that macrophage RhoA deletion promotes chondrocyte senescence via the IL-17C-PI3K-AKT-mTOR signaling axis. Figure 7. IL-17C accelerates chondrocyte senescence via PI3K/AKT/mTOR activation. A. WikiPathways enrichment analysis of RNA-seq data from chondrocytes co-cultured with macrophages. B. WB analysis of PI3K,

AKT, mTOR, p-AKT, and p-mTOR in co-cultured chondrocytes. C-F. WB and SA-β-gal assays quantifying LC3B, iNOS, MMP13, p53, p21, and senescent mouse chondrocytes. G, H. IF analysis of iNOS and ROS in human OA chondrocytes. I, J. WB and SA-β-gal assays quantifying p53, p21, and senescent human OA chondrocytes. *P 0.05. 8. RhoA regulates IL-17C expression in macrophages via the LATS/YAP/CCN2 axis. To elucidate how macrophage RhoA regulates OA progression, we first examined its canonical downstream effector ROCK. WB analysis showed that macrophage-specific RhoA deletion did not significantly alter ROCK expression in macrophages (Fig. 8A), suggesting that RhoA acts independently of ROCK in this context. RNA-seq of primary macrophages from control and cKO mice revealed that RhoA deletion upregulated 1775 genes and downregulated 444 genes (Fig. 8 B-D). KEGG analysis of differentially expressed genes identified the top 20 enriched pathways, with literature review highlighting a correlation between the Hippo signaling pathway, OA progression, and the IL-17 pathway (Fig. 8E). WB analysis showed a significant upregulation of phosphorylated LATS (p-LATS), phosphorylated YAP (p-YAP), together with their downstream target CCN2, indicating Hippo pathway activation in RhoA-deleted macrophages. Treatment with the Hippo pathway inhibitor XMU-MP-1 (400μM)(Li et al. 2024) significantly reduced IL-17C protein expression and secretion in cKO macrophages, as shown by WB analysis and ELISA (Fig. 8 F-J). Co-culture of human OA chondrocyte explants with XMU-MP-1-treated cKO macrophages resulted in reduced matrix loss compared to untreated cKO macrophages (Fig. 8K). These results suggest that RhoA deletion in macrophages promotes IL-17C secretion and exacerbates OA progression via activation of the LATS/YAP/CCN2 signaling axis. Figure 8: Macrophage RhoA regulates IL-17C secretion via the LATS/YAP/CCN2 signaling axis. A. WB analysis of ROCK protein levels in macrophages. B-F. RNA-seq analysis of Cre vs. cKO macrophages. B. Volcano plot of differentially expressed genes (DEGs). C. Heatmap of DEGs across samples. D, E. KEGG enrichment analysis of DEGs. F-I. WB analysis of LATS, YAP, their phosphorylated forms, CCN2 and IL-17C in macrophages. J. ELISA of IL-17C in macrophage supernatants. K. Safranin O and Toluidine Blue staining assessing matrix loss in human OA cartilage explants. *P 0.05. N.S.: not significant. Discussion This study reveals a novel and critical role for macrophage-specific RhoA signaling in alleviating OA progression, which is distinct from its reported roles in chondrocytes or endothelial cells in OA pathogenesis(He et al. 2024; Jiang et al. 2023). While previous literature has firmly established RhoA/ROCK as a promoter of cartilage catabolism and subchondral bone pathology(Yang et al. 2023; Zhu et al. 2013), our findings paradoxically demonstrate that RhoA cKO in the synovial macrophage compartment exacerbates OA disease. This cell-type-specific function underscores the complex, context-dependent nature of RhoA signaling in the joint and highlights the limitations of systemic pharmacological inhibition. As key players in the innate immune response within the joint cavity, synovial macrophages respond to danger-associated molecular patterns, such as necrotic cell debris and cartilage fragments, by releasing cytokines that activate chondrocyte receptor signalling, thereby contributing to OA exacerbation(Chen et al. 2022; Yin et al. 2024; Zhao et al. 2023). This suggests three potential strategies for targeting the detrimental effects of synovial macrophages: (1) blocking the source by elucidating the pathological mechanisms and signalling alterations in synovial macrophages under OA conditions(Blom et al. 2007; Hamasaki et al. 2020; Zhang et al. 2018); (2) targeting the action pathways by identifying and

neutralising secreted pathogenic factors(Liu et al. 2023; Mikulkova et al. 2024); (3) intervening at downstream targets by understanding changes in chondrocyte receptors and signalling pathways to inhibit downstream cascades(Ebata et al. 2021). Present key finding is the identification of IL-17C as the primary effector links the cross-talk of RhoA-deficient macrophages with chondrocyte senescence. The IL-17 family, particularly IL-17A, has been implicated in OA(Faust et al. 2020); however, the role of IL-17C is still unknow. The evidences of current study demonstrate that RhoA acts as a transcriptional brake on IL-17C in macrophages, and its deletion unleashes a cascade of events: heightened IL-17C secretion, activation of the PI3K/AKT/mTOR axis in chondrocytes, suppression of protective autophagy, and ultimately, the induction of a senescent, catabolic chondrocyte phenotype. This macrophage-chondrocyte crosstalk via the RhoA/IL-17C axis represents a significant conceptual advance in understanding OA pathogenesis. Another interesting finding of this study is that RhoA deletion in macrophages activated YAP/CCN2 signaling without altering ROCK expression reveals a non-canonical pathway for RhoA in immune regulation. Previous studies have indicated that RhoA, upon oxidation by mitochondrial superoxide, activates YAP/TAZ, leading to liver damage in injury models(Kwon et al. 2024). It has been reported that CCN2 enhances IL-17 synthesis by disrupting the inhibitory effect of miR655 on IL-17(Zhang et al. 2024). This shifts the paradigm away from the classic RhoA-ROCK cytoskeletal axis and towards the Hippo/YAP pathway as a key regulator of inflammatory cytokine production in OA macrophages. Which suggests that therapeutic strategies targeting RhoA for OA treatment must be highly cell-type-specific, as global inhibition could simultaneously block protective pathways in macrophages and pathogenic pathways in other joint cells. For example, Allen et al. demonstrated that opposing RhoA actions in spinal neurons versus astrocytes restrict regeneration; only neuron-specific RhoA inhibition or blockade of its astrocyte-proliferative effectors fully unleashes the pathway’ s repair potential(Stern et al. 2021). Second, this study indicates IL-17C as a promising and more tractable therapeutic target. The synergistic benefit observed when combining a RhoA activator (CT04) with an IL-17C-neutralizing antibody provides a compelling preclinical rationale for a multi-targeted approach to mitigate macrophage-driven joint destruction. In conclusion, our results demonstrate a previously unknown RhoA/YAP/IL-17C signaling axis in synovial macrophages that serves as a critical pathway against OA progression. By IL-17C paracrine, macrophage RhoA plays important role in maintaining chondrocyte homeostasis and cartilage integrity. This study not only redefines the function of RhoA in OA but also opens new avenues for immunomodulatory therapies aimed at disrupting the pathogenic crosstalk between synovial inflammation and cartilage senescence.
